# Expression profiling and immunolocalization of Na^+^-d-glucose-cotransporter 1 in mice employing knockout mice as specificity control indicate novel locations and differences between mice and rats

**DOI:** 10.1007/s00424-017-2056-1

**Published:** 2017-08-26

**Authors:** Ivana Vrhovac Madunić, Davorka Breljak, Dean Karaica, Hermann Koepsell, Ivan Sabolić

**Affiliations:** 1 Molecular Toxicology Unit, Institute for Medical Research and Occupational Health, Ksaverska cesta 2, 10000 Zagreb, Croatia; 2 Department of Molecular Plant Physiology and Biophysics, University of Würzburg, Würzburg, Germany

**Keywords:** Sodium-d-glucose cotransporter, SGLT1, Mouse, Tissue distribution, Localization, Immunocytochemistry, Nonspecific antibody binding, mRNA expression, Sex differences

## Abstract

The expression and localization of sodium-d-glucose cotransporter SGLT1 (*SLC5A1*), which is involved in small intestinal glucose absorption and renal glucose reabsorption, is of high biomedical relevance because SGLT1 inhibitors are currently tested for antidiabetic therapy. In human and rat organs, detailed expression profiling of SGLT1/Sglt1 mRNA and immunolocalization of the transporter protein has been performed. Using polyspecific antibodies and preabsorption with antigenic peptide as specificity control, in several organs, different immunolocalizations of SGLT1/Sglt1 between human and rat were obtained. Because the preabsorption control does not exclude cross-reactivity with similar epitopes, some localizations remained ambiguous. In the present study, we performed an immunocytochemical localization of Sglt1 in various organs of mice. Specificities of the immunoreactions were evaluated using antibody preabsorption with the Sglt1 peptide and the respective organs of *Sglt1* knockout mice. Because staining in some locations was abolished after antibody preabsorption but remained in the knockout mice, missing staining in knockout mice was used as specificity criterion. The immunolocalization in mouse was identical or similar to rat in many organs, including small intestine, liver, and kidney. However, the male-dominant renal Sglt1 protein expression in mice differed from the female-dominant expression in rats, and localization in lung, heart, and brain observed in rats was not detected in mice. In mice, several novel locations of Sglt1, e.g., in eyes, tongue epithelial cells, pancreatic ducts, prostate, and periurethral glands were detected. Using end-point and quantitative RT-PCR in various organs, different Sglt1 expression in mice and rats was confirmed.

**Electronic supplementary material:**

The online version of this article (doi:10.1007/s00424-017-2056-1) contains supplementary material, which is available to authorized users.

## Introduction

In mammals, d-glucose absorption across epithelial cells is accomplished by the coordinated action of the Na^+^-d-glucose cotransporters SGLT1 or SGLT2 in the luminal membrane and the facilitative d-glucose transporters in the contraluminal membrane [15, 27, 31]. The SGLT transporters belong to the *SLC5* family and the GLUT transporters to the *SLC2* family. Initially, studies on tissue expression and distribution of SGLT1 (Sglt1 in rodents) and SGLT2 (Sglt2 in rodents) were based mainly on messenger RNA (mRNA) analysis [14, 27, 31, 61]. The data indicated that SGLT1/Sglt1 is predominantly expressed in small intestine and kidney but also in numerous other organs, whereas SGLT2/Sglt2 is more specifically expressed in kidney ([31] and references therein). Due to the low quality and equivocal specificity of first-generation antibodies, the protein distribution and localization of SGLT1/Sglt1 was initially determined mainly in small intestine and kidney where the transporter is highly expressed [24, 25, 27, 49, 50].

Starting 20 years ago, we generated polyclonal antibodies against peptides of SGLT1/Sglt1 from human, rat, and mouse and characterized their immunoreactivity by immunocytochemistry and Western blot analysis using absorption of the antibodies with their respective antigenic peptides and selectivities for SGLT/Sglt subtypes as controls for antibody specificity [2, 18, 19, 21, 30, 41, 45, 46, 54]. In rat and human, we investigated the distributions of Sglt1/SGLT1 in small intestine and kidney in detail, including gender dependence. Moreover, we analyzed immunoreactivity of the antibodies in additional organs and tissues such as brain, heart, and skeletal muscle. So far, we only communicated a few immunocytochemical data from mice concerning selected regions of small intestine and kidney [21]. In human and rat SGLT1/Sglt1, immunoreactivity was observed in many identical locations, e.g., brush-border membrane (BBM) of enterocytes, BBM in S3 segments of renal proximal tubules, luminal membrane of biliary ducts in the liver, small vessels in heart, and in alveolar type 2 epithelial cells and bronchiolar Clara cells in lungs [2, 19, 54]. In addition, several distinctly different sites of immunoreactivity between human and rat were observed. For example, in rat, but not in human, immunoreactivity was detected in brain neurons and thick ascending limb of Henle (TALH) including the macula densa. The up-to-now described localizations of SGLT1/Sglt1 in human and animal organs have been reviewed recently [31].

The present detailed immunolocalization of Sglt1 in intestine, kidney, and various additional organs and tissues of mice was performed for two reasons. First, the preabsorption specificity controls, performed in our previous immunolocalization studies in human and rat, do not unequivocally exclude false-positive localizations of SGLT1/Sglt1 which may be due to the cross-reactivity of the antibodies with similar epitopes that did not show up during sequence comparison of the antigenic peptides since they may be conformational in nature [11, 37]. Because we had generated the *Sglt1* knockout mouse (*mSglt1* ^*−/−*^), optimal specificity controls of antibody reactions in different tissues could be performed so that cross-reactivity could be excluded unequivocally. Second, differences between expression of some transporters in mice and rats have been described [44], and the use of SGLT1 inhibitors for antidiabetic therapy is currently investigated using these species ([31, 60] and references in there). Thus, to analyze potential side effects of SGLT1 inhibitors due to inhibition of SGLT1 expressed in locations apart from small intestine and kidney, it has to be known whether rats and mice express Sglt1 in the same locations and can be equally used for preclinical testing relevant for humans.

**Fig. 1:**
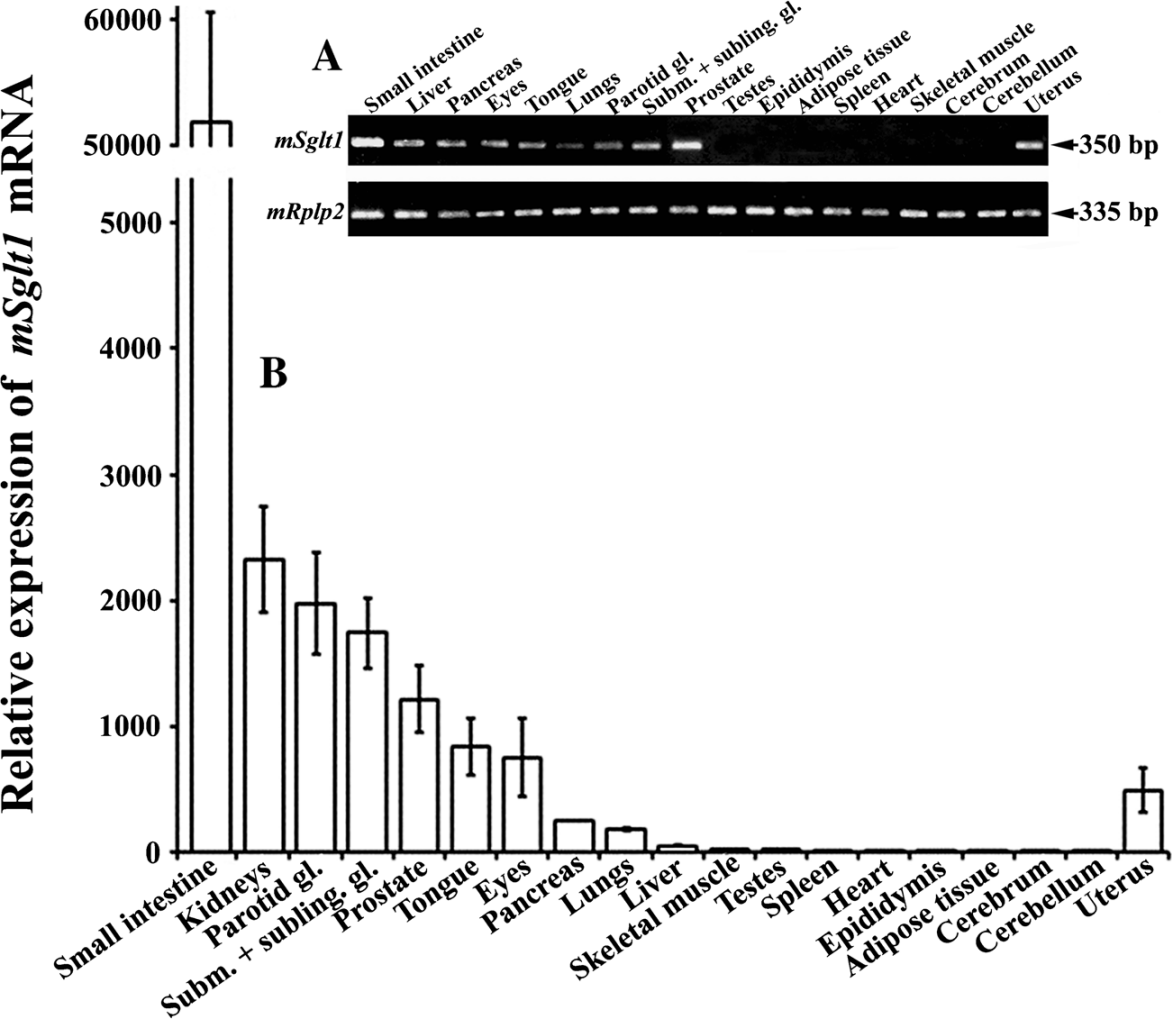
Relative expression of *mSglt1* mRNA in various organs/tissues of wild-type mice estimated by end-point RT-PCR and qRT-PCR. **a** End-point RT-PCR. The *mSglt1*-related PCR product of 350 bp and *mRplp2*-related PCR product of 335 bp (housekeeping gene) after 30 PCR cycles in various organs/tissues. Data are representative for similar findings in the organs/tissues from three male and three female wild-type animals. In *mSglt1* ^*−/−*^ animals, the *mSglt1* mRNA expression was not observed in any organ (data not shown). **b** qRT-PCR. The mRNA expression data are presented relatively to the lowest *mSglt1* mRNA levels measured in cerebellum. Data (mean ± SEM) were obtained with cDNA samples prepared from three males, except for uterus, obtained from three females. The mRNA expression of housekeeping gene *mHprt* was similar in all tested RNA samples (data not shown). Subm. + subling. gl., combined tissue from submandibular and sublingual glands

**Fig. 2:**
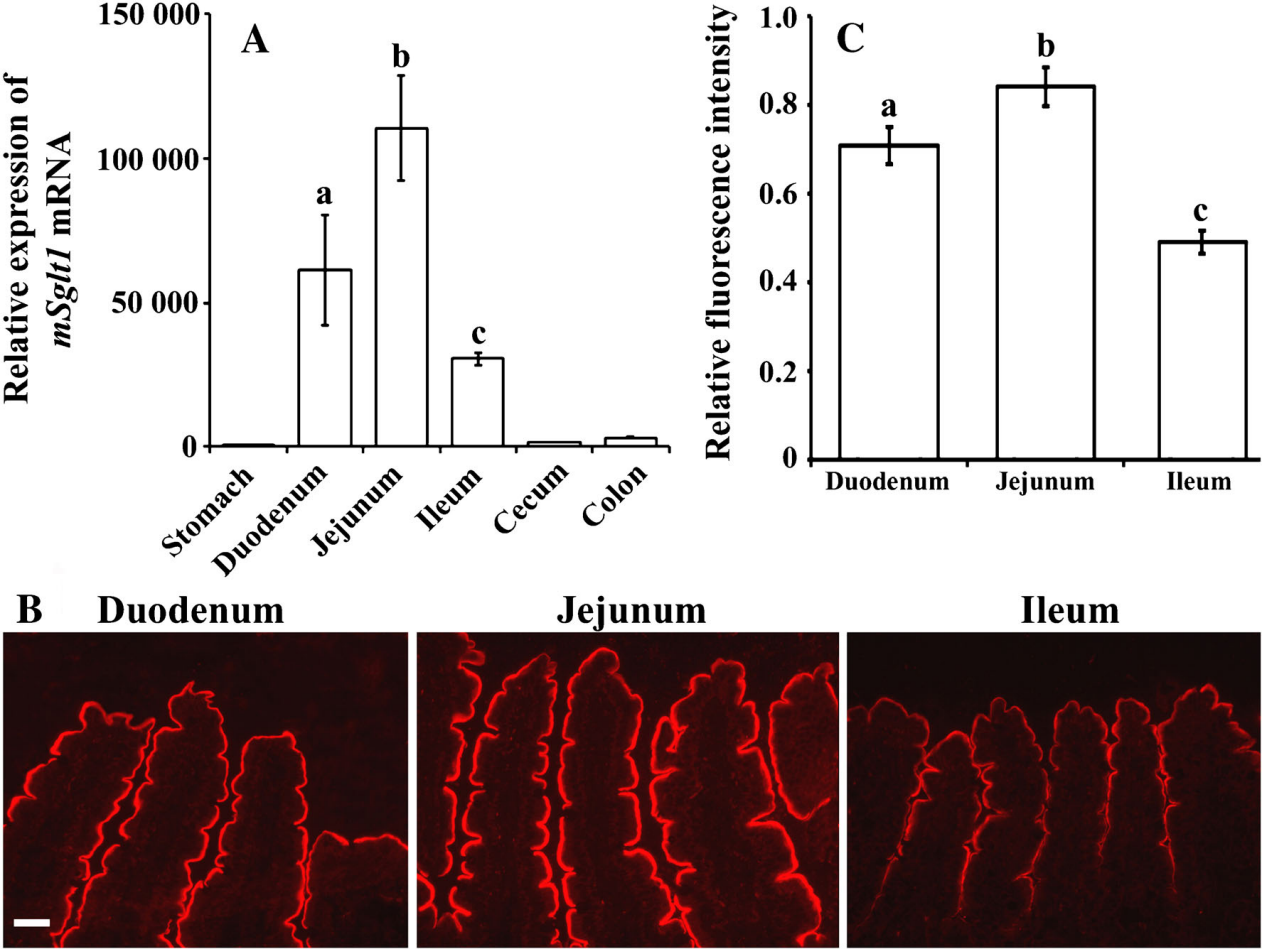
Expression of *mSglt1* mRNA (**a**) and mSglt1 protein (**b**, **c**) in the gastrointestinal tract of wild-type mice. **a** The expression levels of *mSglt1* mRNA in various segments of gastrointestinal tract, as determined by qRT-PCR. The results in specific segments are presented relative to that in cerebellum, where the lowest mRNA concentration was measured (c.f. the data in Fig. 1b). Data are means ± SEM obtained from four male animals. Statistics (ANOVA/Duncan), *P* < 0.05: a vs. b or c, and b vs. c. **b** mSglt1-Ab stained the enterocyte BBM in small intestine with the segment-specific intensity (jejunum > duodenum > ileum). No staining was detected in stomach, cecum, and colon (data not shown). Bar, 20 μm for all images. **c** The segment-specific intensity of fluorescence staining in small intestine (relative to that in jejunum) was determined in cryosections from three males and three females. From each animal, six regions from two images were analyzed, and the results were combined, averaged, and used as a single datum. Each bar represents mean ± SEM of the data from six animals. Statistics (ANOVA/Duncan), *P* < 0.05: a vs. b or c and b vs. c

**Fig. 3:**
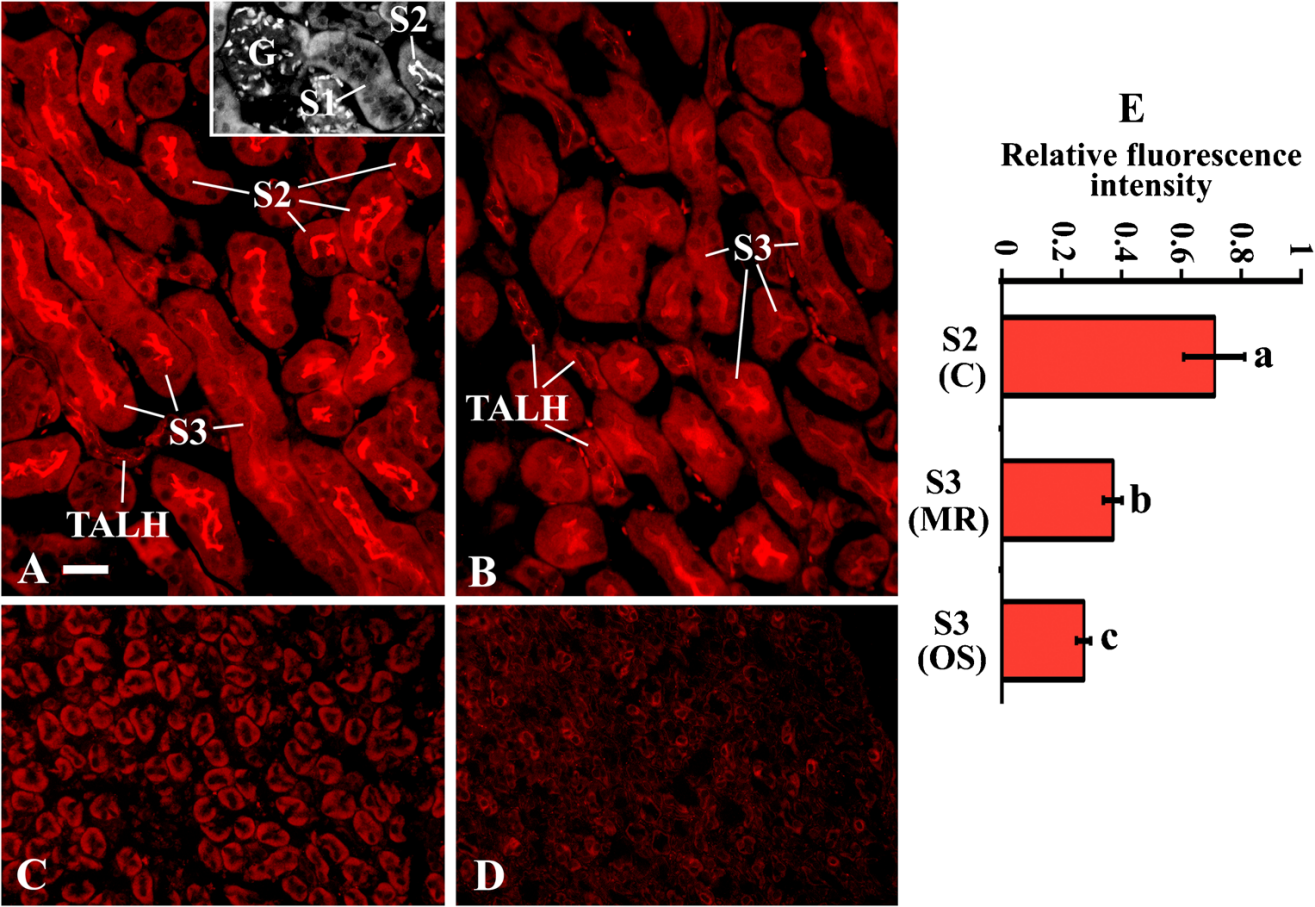
Immunostaining of the nephron segments in various kidney zones of male wild-type mice with mSglt1-Ab. **a** Cortex. No staining was observed in glomeruli; S1 segments (inset), distal tubules and cortical collecting ducts (not shown) were not stained. The luminal domains of S2 segments, S3 segments in medullary rays, and TALH were stained with variable intensity. **b** Outer stripe. The luminal domain of S3 segments and TALH was stained with variable intensity; the terminal part of S3 segments was stained stronger than the proximal part. **c** Inner stripe, and **d** Inner medulla/papilla. TALH and other structures were not stained. Bar, 20 μm for all images. **e** The relative fluorescence intensity of the BBM staining in proximal tubule S2 and S3 segments. In each animal, 8–10 ROI from two images were analyzed; the results were combined, averaged, and used as a single datum. The results are expressed relatively to the strongest intensity measured in the BBM of S2 segments. *C* cortex, *MR* medullary rays, *OS* outer stripe. Each bar represents mean ± SEM of data measured in four mice. Statistics (ANOVA/Duncan), *P* < 0.05: **a** vs. **b** or **c** and **b** vs. **c**

**Fig. 4:**
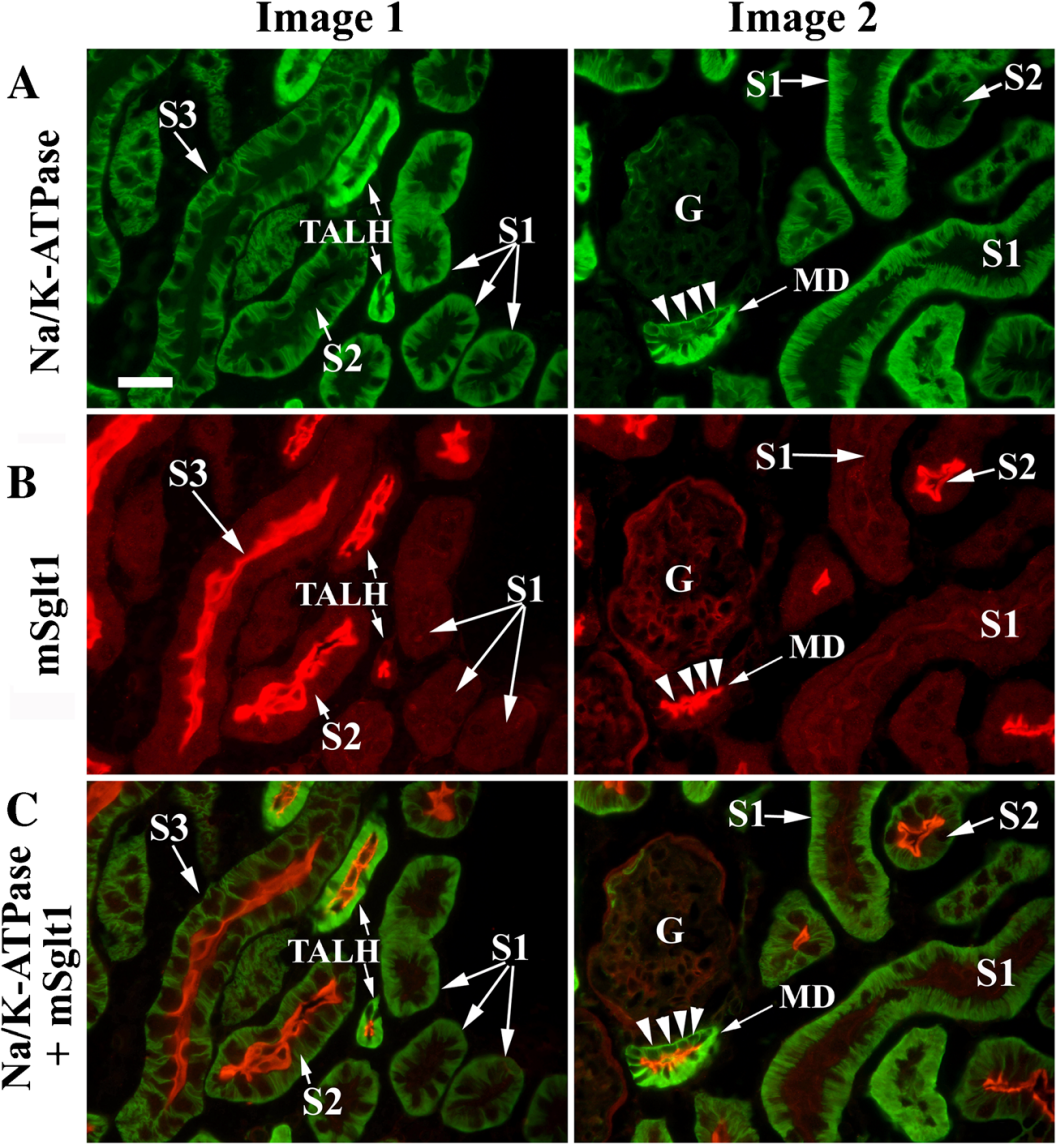
Double immunostaining of kidney cortex of wild-type mice with mSglt1-Ab (red fluorescence) and Na/K-ATPase-Ab (green fluorescence) confirms the localization of mSglt1 in TALH and macula densa (MD). **a** Na/K-ATPase-Ab stained the BLM; moderately in proximal tubule S1, S2, and S3 segments; and strongly in TALH and MD. **b** mSglt1-Ab stained the BBM of S2 segments and the luminal membrane of TALH and MD. At the luminal membrane of S1 segments, mostly no Sglt1-Ab immunoreactivity was detected (Image 1); however, sometimes, a very weak staining was observed (Image 2). In the merged images (Na/K-ATPase + mSglt1), the colocalization of Na/K-ATPase and mSglt1 in the basolateral and luminal membranes of specific nephron segments can be seen. The very strong expression of the Na/K-ATPase is typical for TALH and macula densa. *G* glomerulus. Bar, 20 μm for all images

**Fig. 5:**
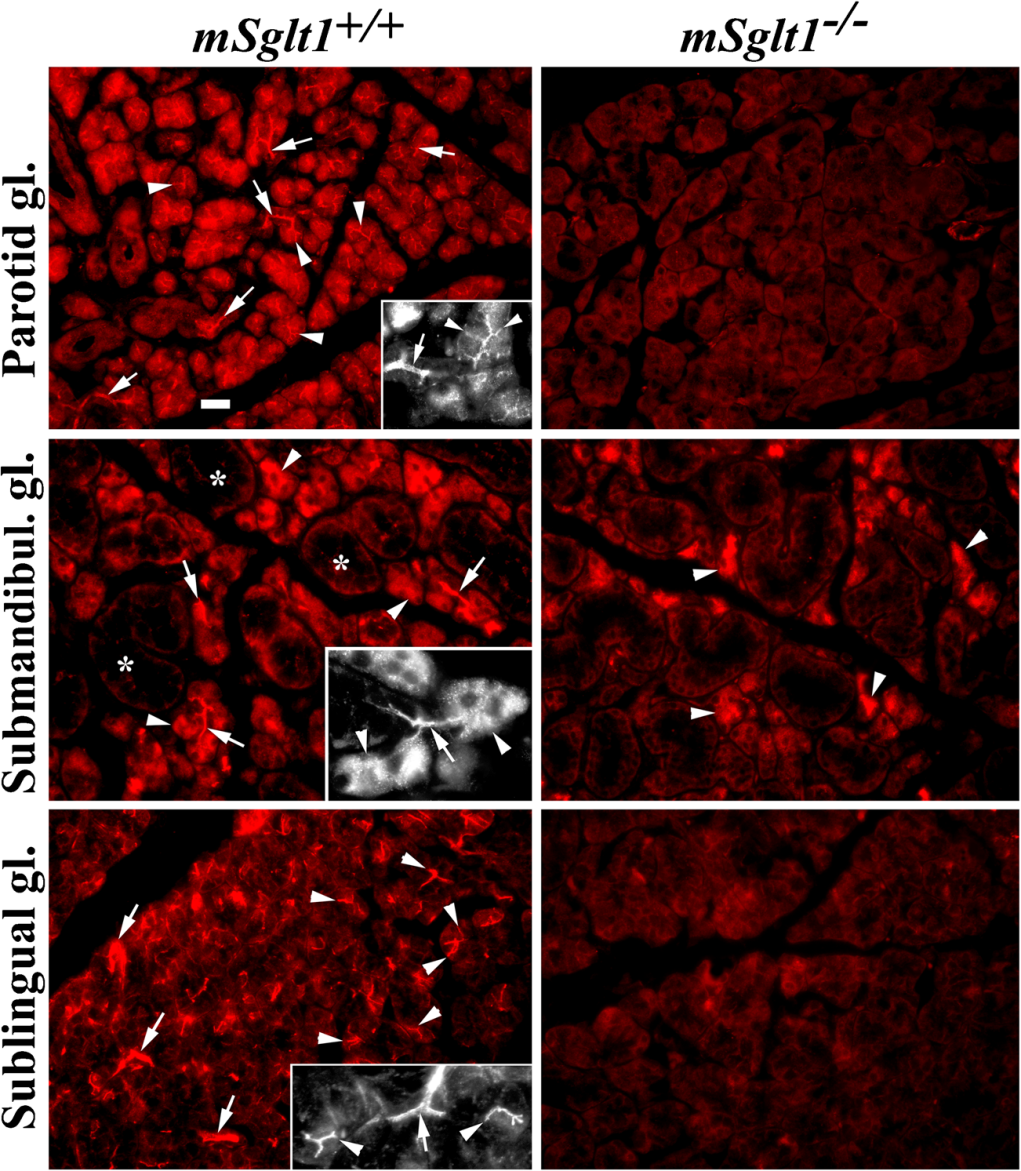
Immunolocalization of mSglt1 protein in salivary glands from *mSglt1* ^*+/+*^ and *mSglt1* ^*−/−*^ mice. Parotid gland. In *mSglt1* ^*+/+*^ mice (main image and inset), mSglt1-Ab stained the apical membrane of acinar cells (arrowheads) and initial ducts (arrows) of this serous gland. In *mSglt1* ^*−/−*^ mice, this staining was not observed. Submandibular gland. In *mSglt1* ^*+/+*^ mice (main image and inset), the mSglt1-Ab stained the initial ducts of serous acini (arrows) and intracellular granule-like organelles in many acinar cells (arrowheads) of this seromucous gland. The mucous acini remained unstained (asterisks). In *mSglt1* ^*−/−*^ mice, the staining of initial ducts was absent, but the intracellular granular staining of variable intensity remained, indicating cross-reactivity with an intracellular protein (arrowheads). Sublingual gland. In *mSglt1* ^*+/+*^ mice (main image and inset), the apical membrane of the acinar cells (arrowheads) and the initial segments of the salivary ducts of this mucoserous gland (arrows) are stained. In *mSgtl1* ^*−/−*^ mice, the staining of acinar cells and initial ducts was absent. mSglt1-Ab immunostaining in all locations was abolished when peptide-blocked antibody was used (data not shown). Bar, 20 μm for all images

**Fig. 6:**
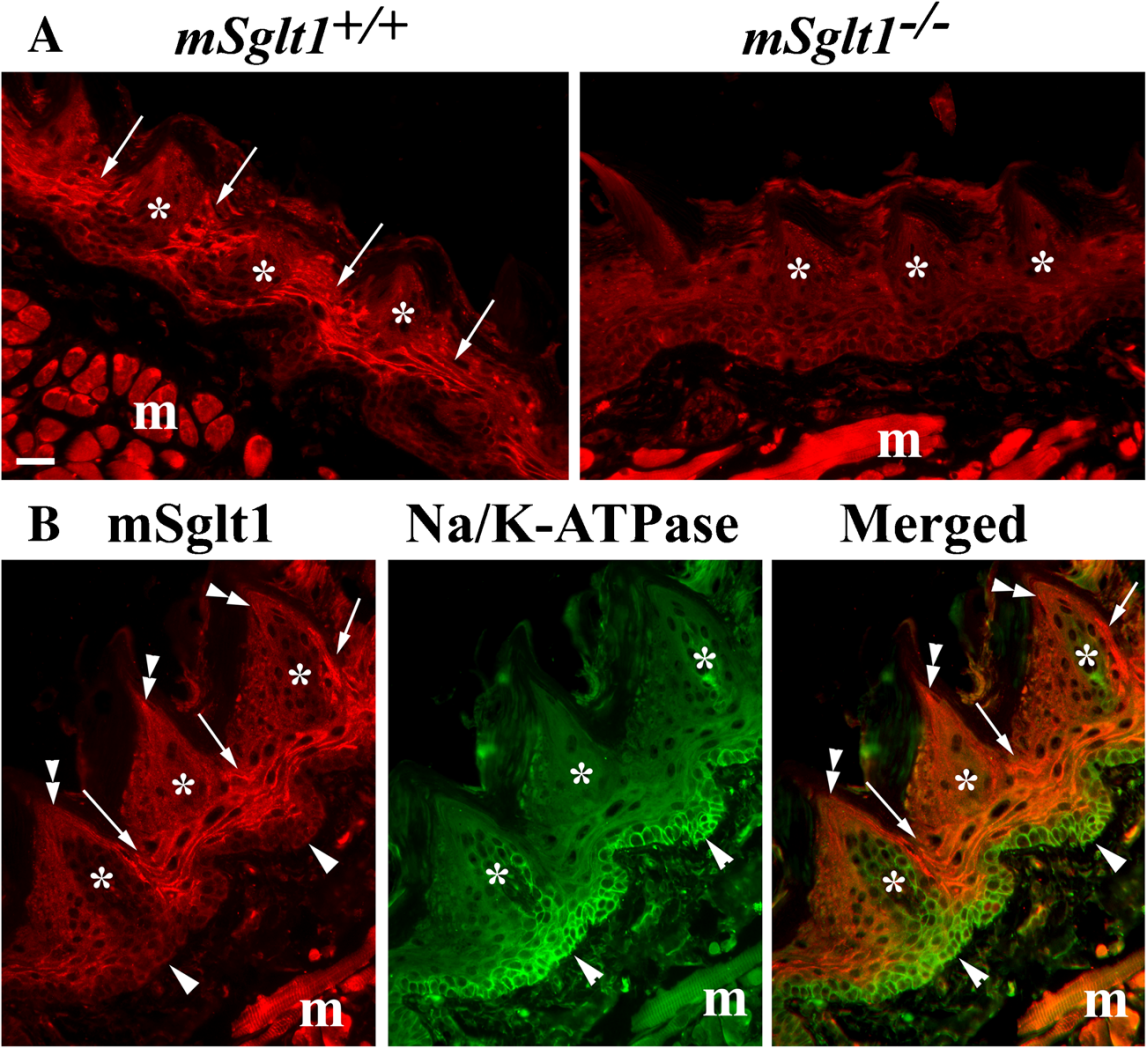
Immunolocation of Sglt1 in the tongue of *mSglt1* ^*+/+*^ and *mSglt1* ^*−/−*^ mice; co-staining with Na/K-ATPase-Ab. **a** In *mSglt1* ^*+/+*^ mice, the plasma membrane of epithelial cells (arrows) surrounding the taste buds were strongly stained (asterisks). This staining was absent in *mSglt1* ^*−/−*^ mice. The immunostaining of tongue muscles (m) was similar in both wild-type and *mSglt1* ^*−/−*^ mice but not observed with peptide-blocked antibody (data not shown) indicating non-specificity. **b** Double immunostaining with mSglt1-Ab (red) and Na/K-ATPase-Ab (green) differentiates between plasma membrane staining of epithelial cells for mSglt1, which was observed in the cells surrounding the lateral and bottom sides of the taste buds (arrows) and plasma membrane staining of basal epithelial cells (arrowheads) and of cells within the taste buds (asterisks) for Na^+^/K^+^-ATPase. Staining of the tips of longitudinal sectioned taste buds with mSglt1-Ab suggests the location of mSglt1 in cilia of taste cells (double arrowheads). *m* skeletal muscles. Bar, 20 μm for all images

**Fig. 7:**
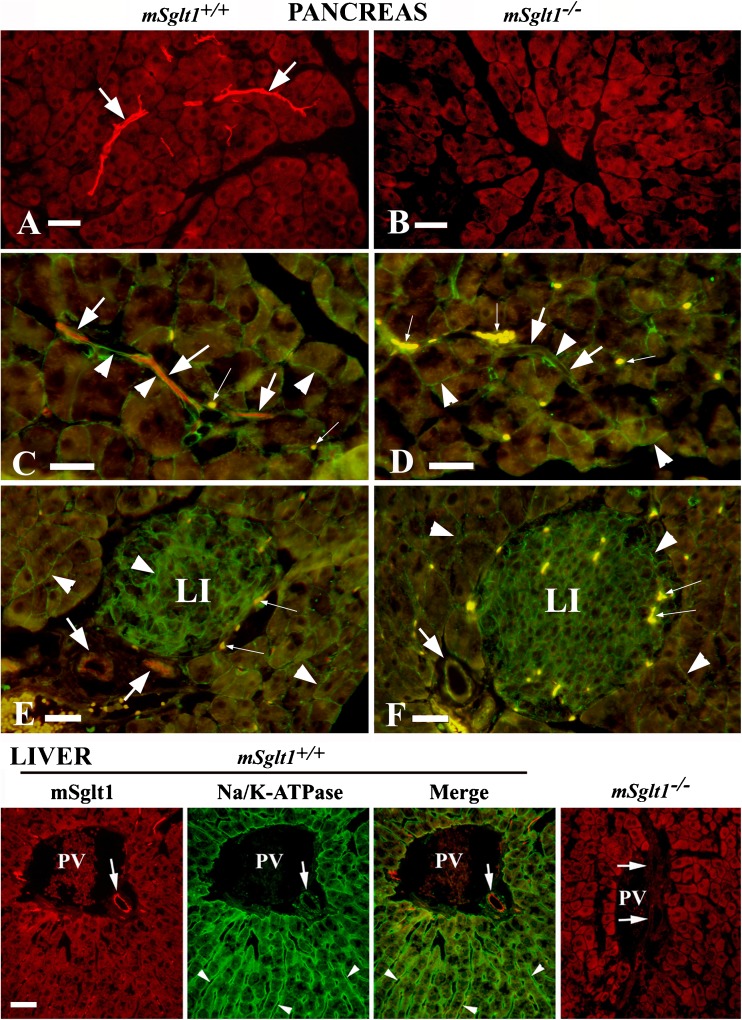
Immunolocation of Sglt1 in pancreas and liver with mSglt1-Ab in *mSglt1* ^*+/+*^ and *mSglt1* ^*−/−*^ mice and co-staining with the Na^+^/K^+^-ATPase. Pancreas (**a**, **b**). mSglt1-Ab stained the luminal membrane of intralobular ducts (arrows) in *mSglt1* ^*+/+*^, but not in *mSglt1* ^*−/−*^ mice. The staining in *Sglt1* ^+/+^mice was also absent with the antibody that had been preabsorbed with the immunizing peptide (not shown). **c**–**f** Co-staining with mSglt1-Ab (red) and Na/K-ATPase-Ab (green). In *mSglt1* ^*+/+*^ animals (**c**, **e**), mSglt1-Ab immunoreactivity was observed in the luminal domain of intralobular (**c**) and interlobular (**e**) ducts (arrows, red fluorescence), whereas Na/K-ATPase-Ab stained the plasma membrane of acinar cells, Langerhans islet cells (LI), and the BLM of intralobular (**c**) and interlobular (**e**) duct cells (arrowheads, green fluorescence). In *mSglt1* ^*−/−*^ mice, no staining of ducts with mSglt1-Ab was observed (arrows in **d**, **f**). In Langerhans islets of both *mSglt1* ^*+/+*^ and *mSglt1* ^*−/−*^ mice, no immunostaining with mSglt1-Ab related was observed (**e**, **f**). Autofluorescence of red blood cells is indicated by thin arrows. Bars, 20 μm. Liver. In *mSglt1* ^*+/+*^ animals, co-staining with mSglt1-Ab and Na/K-ATPase-Ab showed the presence of mSglt1 protein in the luminal domain of bile ducts (mSglt1 and Merge, arrow), whereas Na/K-ATPase-Ab stained the sinusoidal membrane of hepatocytes (arrowheads) and the BLM of bile duct epithelium (Na/K-ATPase and Merge, arrow). The mSglt1-Ab-related staining of the bile ducts was absent in the liver of *mSglt1* ^*−/−*^ mice and also not observed with antibody that had been preabsorbed with the immunizing peptide (not shown). In hepatocytes, some non-specific background staining was observed. *PV* portal vein; Bar, 20 μm for all images

**Fig. 8:**
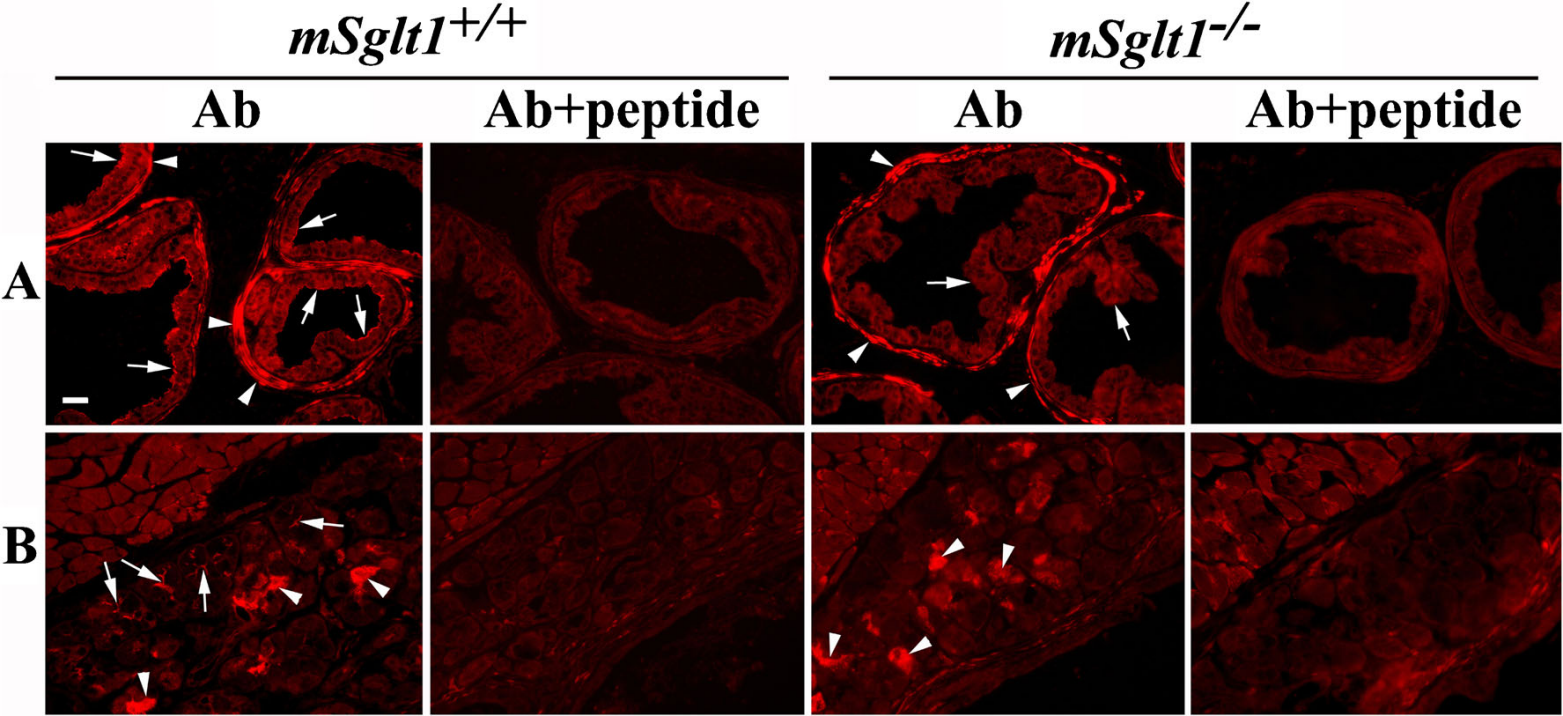
Immunolocalization of mSglt1 protein in prostate and periurethral glands. **a** Prostate. In *mSglt1* ^*+/+*^ mice (Ab), mSglt1-Ab stained the luminal membrane of glandular epithelial cells (arrows) and myoepithelial cells that surround the glands (arrowheads). Staining in both locations was abolished when mSglt1-Ab had been preabsorbed with the immunizing peptide (Ab + peptide). In *mSglt1* ^*−/−*^ mice, the luminal membrane of the glandular epithelium was unstained (Ab, arrows) whereas staining of myoepithelial cells was unchanged (Ab, arrowheads). At variance, staining in both locations was abolished after preabsorption of the antibody with immunizing peptide (Ab + peptide), indicating cross-reactivity with myoepithelial cells. **b** Periurethral gland. In *mSglt1* ^*+/+*^ mice, mSglt1-Ab stained the apical membrane of acinar cells (Ab arrows) and an intracellular granular material (Ab, arrowheads). After preabsorption of mSglt1-Ab with antigenic peptide (Ab + peptide), in *mSglt1* ^*+/+*^ mice, no staining was observed in both locations. In *mSglt1* ^*−/−*^ mice (Ab), the apical membrane of acinar cells was not stained, whereas the intracellular staining remained unchanged (arrowheads) but abolished with mSglt1-Ab that had been preabsorbed with the immunizing peptide (Ab + peptide). This indicates cross-reactivity with an intracellular protein

**Fig. 9:**
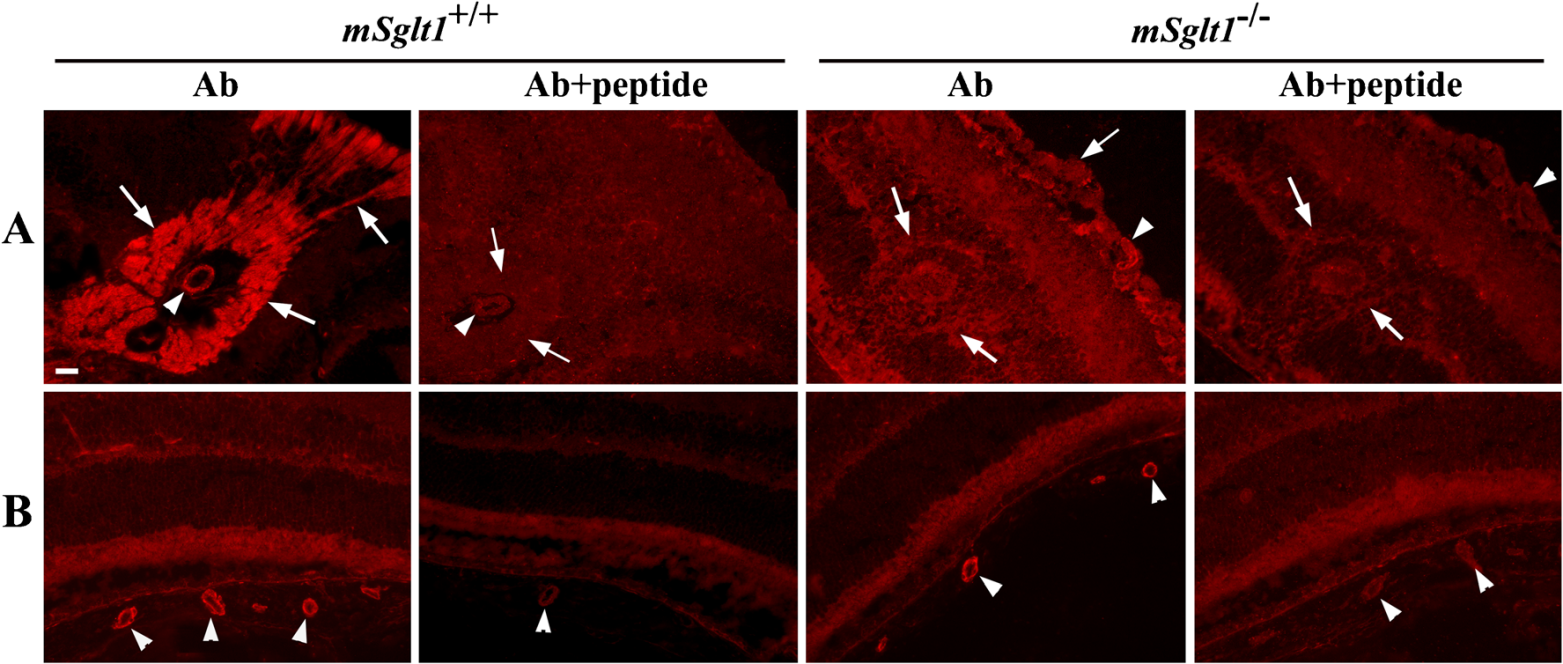
Immunolocalization of mSglt1 protein in tissue cryosections of eyes from *mSglt1* ^*+/+*^ and *mSglt1* ^*−/−*^ mice. In *mSglt1* ^*+/+*^ mice, mSglt1-Ab strongly stained the axons from ganglion cells and optic nerve (**a**, Ab, arrows), and weaker the wall of central artery in the optic nerve (**a**, Ab, arrowhead) and the arterial wall in retinal periphery (**b**, Ab, arrowheads). In *mSglt1* ^*−/−*^ mice, the staining in axons and optic nerve was absent (Ab, arrows), whereas the staining of arterial walls remained (Ab, arrowheads). In both *mSglt1* ^*+/+*^ and *mSglt1* ^*−/−*^ mice, the mSglt1-Ab-related staining in all locations was abolished when mSglt1-Ab had been preabsorbed with immunizing peptide (**a**, **b**, Ab + peptide). Bar, 20 μm for all images

**Fig. 10:**
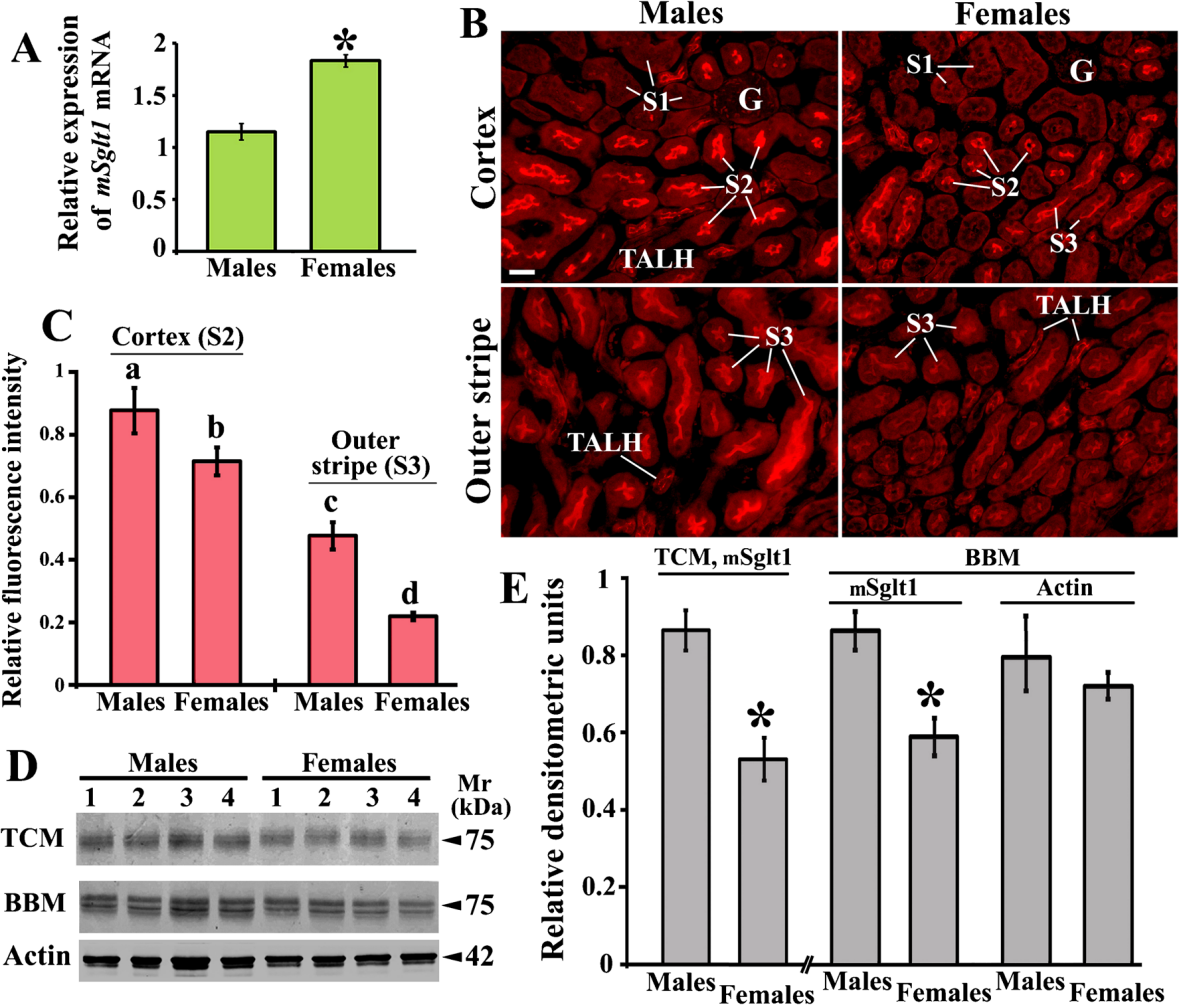
Sex-related expression of *mSglt1* mRNA and mSglt1 protein in the kidneys of wild-type mice. **a** In qRT-PCR, relative expression of *mSglt1* mRNA in females was stronger than in males. In both sexes, a similar expression of *mHprt* mRNA was observed (data not shown). Each bar represents the data (mean ± SEM) obtained from measurements (two per animal) in four animals. *P < 0.05 vs. males. **b** mSglt1-Ab-related immunostaining in the cortex and outer stripe from male and female kidneys. *G* glomeruli; S1, S2, and S3, proximal tubule segments. TALH thick ascending limb of Henle loop. Bar, 20 μm for all images. **c** Fluorescence staining intensity of the BBM in S2 (cortex) and S3 (outer stripe) segments was stronger in males (~ 20% in cortex and ~ 130% in outer stripe). In each animal, 8–10 ROI from two images were analyzed; the results were combined, averaged, and used as a single datum. Shown are means ± SEM of the data for four animals in each group. Statistics (ANOVA/Duncan), *P* < 0.05: a vs. b, c or d; b vs. c or d; c vs. d. **d** Western blots of TCM and BBM, isolated from the whole kidneys from male and female mice, were blotted with mSglt1-Ab (TCM and BBM) and actin-Ab (BBM). Each band represents isolated membranes (40 μg protein/lane) from a separate animal. Male-dominant mSglt1 protein bands, but sex-independent actin protein bands, were observed. **e** Densitometric quantification of the bands shown in **d**. The density of mSglt1-related ~ 75 kDa protein bands in TCM and BBM was ~ 60 and 33%, respectively, stronger in males than in females. The density of actin bands was similar in both sexes. Shown are means ± SEM obtained with membrane preparations from four male and four female mice. *P < 0.05 vs. males

In the manuscript, the following data are presented: First, we described the expression of *mSglt1* mRNA in various organs using the *Sglt1* knockout mice as controls (Figs. 1 and S1). Second, we characterized our non-commercial polyclonal antibody mSglt1-Ab which was used for immunolocalization of mSglt1 protein in mouse organs (Fig. S2). Third, we described the specific immunolocalization of mSglt1 protein in intestine, kidney, and in various other mouse organs employing this antibody using tissues from *mSglt1* ^−/−^ mice as controls (Figs. 2, 3, 4, 5, 6, 7, 8, and 9). For immunolocalization of Sglt1 in several mouse organs, fundamental differences were observed compared to rat and some new sites of expression were detected. Fourth, we presented data which indicate that also the sex differences that have been previously described in rat kidney are diverging in mice (Fig. 10). Fifth, we presented a site-by-site comparison of quantitative mRNA analyses in several mouse and rat organs which indicated distinct differences in some organs (Table 2). Finally, experimental data are reported in which we tried to elucidate why various diverging immunolocalizations of Sglt1 in mice have been reported in the literature. We presented data showing missing specific immunoreactivity for mSglt1-Ab in lung, heart, and brain (Figs. S3, S4, and S5). In addition, we characterized immunostaining with three commercial antibodies in several locations including lung, heart, and brain using tissues from *Sglt1* ^−/−^ mice as controls (Figs. S6, S7, S8, S9, S10, and S11).

Taking together, our data provide a reevaluation and extension of knowledge concerning the expression of Sglt1 in mouse. The data are supposed to provoke more critical interpretations of immunoreactivity of antibodies raised against SGLT1/Sglt1 in immunocytochemistry.

## Materials and methods

### Animals

Three- to 5-months-old wild-type C57BL/6 mice (*mSglt1* ^*+/+*^) and *mSglt1* knockout mice of the same background (*mSglt1* ^*−/−*^) of both sexes were obtained from the central animal facility of University of Würzburg (Germany). Generation of the *mSglt1* ^−/−^ mice was described earlier [21]. These animals were used for all experiments with the exception of those reported in Table 2 and Fig. 10. For mRNA profiling by end-point and quantitative RT-PCR (qRT-PCR), and for protein profiling by immunocytochemistry shown in Figs. 1, 2, 3, 4, 5, 6, 7, 8, and 9 and Figs. S1, S2, S3, S4, S5, S6, S7, S8, S9, S10, and S11, each organ or tissue was sampled from three males and three females. The sex-related mRNA and immunochemical studies were performed in the organs/tissues from four adult male and four female C57BL/6 strain mice that were purchased from the breeding colony at the Rudjer Boskovic Institute (IRB) in Zagreb, Croatia. To compare the expression of *Sglt1* mRNA in various organs between mouse and rat, presented in Table 2, samples from three adult male wild-type C57BL/6 mice and three adult Wistar strain male rats were used. The mice were purchased from the IRB, and the rats were from the Institute for Medical Research and Occupational Health (IMROH) in Zagreb, Croatia. The experiments were approved by the Ethics Committee of the IMROH in Zagreb and were conducted in accordance with the Directive 2010/63/EU on the protection of animals used for scientific purposes.

### RNA extraction and cDNA synthesis

Mice were sacrificed by cervical dislocation and rats by decapitation, their abdominal cavities were opened, and large blood vessels were cut and let to bleed out under a stream of cold running water. Tissues from various organs, including adipose tissue (abdominal fat), brain (cerebrum, cerebellum), eyes, heart, kidneys, liver, lungs, gastrointestinal segments (stomach, duodenum, jejunum, ileum, cecum, colon), male reproductive tract (testes, epididymides, prostate), pancreas, salivary glands (parotid, submandibular, sublingual), skeletal muscle (*M. quadriceps*), spleen, tongue, and uterus were sampled, immediately submerged in RNAlater solution (Sigma, USA), and stored at − 20 °C until use. Total cellular RNA from the sampled tissues was extracted and purified using TRIzol® Plus RNA Purification Kit (Ambion, Life Technologies, USA) according to manufacturer’s instructions. RNA concentrations and purity were determined spectrophotometrically (BioSpec Nano, Shimadzu, Japan). RNA integrity was estimated by agarose gel electrophoresis, staining with ethidium bromide, and visualized under UV light. Isolated RNAs were stored at − 70 °C until use. First-strand complementary DNA (cDNA) was synthesized using the High-Capacity cDNA Reverse Transcription Kit (Applied Biosystems, USA) in accordance with the manufacturer’s guidelines and stored at − 20 °C until use.

### End-point RT-PCR

End-point RT-PCR was performed in 20 μl volume with 1 μl of first-strand cDNA (100 ng), PCR buffer (1×), dNTP mix (0.2 mM), specific primers (0.4 μM), and *AmpliTaq* DNA polymerase (0.025 U/μl) according to manufacturer’s instructions. In order to avoid amplification of genomic DNA, intron spanning primers for *mSglt1* were created by free software Primer 3.0 (v. 0.4.0) (http://bioinfo.ut.ee) and were purchased from Invitrogen. Design of the primers for housekeeping gene mouse ribosomal protein large P2 (*mRplp2*) was previously described [9]. Primer sequences and their predicted RT-PCR product sizes are listed in Table 1. End-point RT-PCR was performed as follows: initial denaturation for 3 min at 94 °C, denaturation for 30 s at 95 °C, annealing for 30 s at 57 °C, and elongation for 45 s at 72 °C. The optimal PCR cycle number was determined experimentally and found to be 30 cycles for both, *mSglt1* and *mRplp2*. To test for possible RT-PCR contamination, the non-template control (NTC) reactions, where the cDNA was substituted with DNase/RNase-free water, were included in each PCR reaction. RT-PCR products were analyzed by electrophoresis in 1.5% agarose gel, stained with ethidium bromide, and visualized under UV light. Subsequently, the respective renal *mSglt1*-related RT-PCR product was excised, purified by QIAquick Gel Purification Kit (QIAGEN, USA) following the manufacturer’s protocol, sequenced by ABI PRISM 3100 Genetic Analyzers (Applied Biosystems, USA) in accordance with the manufacturer’s instructions, and aligned using Nucleotide BLAST (http://blast.ncbi.nih.gov/).

**Table 1:** Primer sequences used for end-point RT-PCR

Gene	Forward (F) and reverse (R) primers (5′ → 3′)	mRNA accession number	Location	RT-PCR product (bp)
*mSglt1*	F: GCCATGGACAGTAGCACCTT	NM_019810.4	236–255	350
	R:AATATCCAGCCCAGCACAAC		566–585	
*mRplp2*	F: TACGTCGCCTCTTACCTGCT	NM_026020.6	66–85	335
	R: AACAAGCCAAATCCCATGTC		381–400	

Accession numbers for *mSglt1* and *mRplp2* mRNAs were identified in public data base (http://www.ncbi.nlm.nih.gov/gene)

### Quantitative real-time RT-PCR

qRT-PCR was performed in 25 μl volume containing 11.25 μl of first-strand cDNA (100 ng), 12.5 μl of the 2× TaqMan Universal PCR Master Mix, and 1.25 μl of 20× TaqMan Gene Expression Assay (all from Applied Biosystems, USA). IDs for TaqMan Gene Expression Assays were Mm00451203_m1 for the *mSglt1*, Mm00446968_m1 for the mouse housekeeping gene *hypoxanthine guanine phosphoribosyl transferase* (*mHprt*), and Rn01640634_m1 for the *rSglt1* and Rn01527840_m1 for the rat housekeeping gene *rHprt* (www.appliedbiosystem.com).

Amplification and detection were performed using the 7500 Real-Time PCR System (Applied Biosystems). Thermal cycling conditions were preincubation for 2 min at 50 °C and initial denaturation for 10 min at 95 °C, followed by 40 two-step cycles of denaturation (15 s at 95 °C) and annealing/extension (1 min at 60 °C). The NTC reactions were included in every qRT-PCR run in order to detect possible contamination. Each sample was performed in duplicate. The relative quantification of *mSglt1* and *rSglt1* mRNA levels was performed by the comparative Ct-method [34] using Relative Quantification Study Software (Applied Biosystems).

### Antibodies

Non-commercial, affinity-purified rabbit-raised polyclonal antibody against the C-terminal peptide of mSglt1 protein (mSglt1-Ab) was previously described [21]. Commercial polyclonal anti-SGLT1 antibodies were from Merck Milipore (07-1417, Lot no. 2730935), Abcam (ab14686), and Santa Cruz Biotechnology (M-19, sc-20582). According to information in the respective technical sheets, these polyclonal antibodies are supposed to react with the human and rodent SGLT1/Sglt1 proteins. Monoclonal antibodies against actin (actin-Ab) and Na^+^/K^+^-ATPase (Na/K-ATPase-Ab) were purchased commercially from Merck Milipore (MAB1501R) and Santa Cruz Biotechnology (sc-48345), respectively; their use was described in our recent publication [54]. Secondary antibodies included the alkaline phosphatase-labeled goat anti-mouse IgG (GAM-AP) and goat anti-rabbit IgG (GAR-AP) (both from Jackson ImmunoResearch Laboratories, USA) for Western blotting, as well as the CY3-labeled goat anti-rabbit IgG (GAR-CY3) and donkey anti-goat IgG (DAG-CY3) IgG (Jackson ImmunoResearch Laboratories, USA), and FITC-labeled goat anti-mouse IgG (GAM-FITC; Kirkegaard & Perry, USA) for immunocytochemistry.

### Isolation of total cell and brush-border membranes from kidneys and small intestine

Mice were sacrificed by cervical dislocation. The kidneys were removed, decapsulated, rinsed with cold PBS, and minced. The whole gastrointestinal tract was removed; tissue segments (stomach, duodenum, jejunum, ileum, colon) were separated, opened longitudinally, rinsed with cold PBS, and the mucosa was scrapped for further membrane isolation. Renal and gastrointestinal tissue samples were homogenized, and total cell membranes (TCMs) were isolated by differential centrifugation (pellet between 6,000 and 150,000×*g*) as described in detail previously [2, 45, 46]. BBM from the whole kidneys and small intestinal mucosa homogenates were isolated by the Mg^2+^-EGTA precipitation [6]. Protein concentration in TCM and BBM preparations was measured by the Bradford assay [10], and the membranes were stored frozen at − 20 °C until further use.

### SDS-PAGE and Western blotting

Proteins in TCM and BBM were denatured in Laemmli buffer under non-reducing conditions for 15 min at 65 °C, separated by SDS-PAGE mini-gels, and wet-transferred to Immobilon membrane (Milipore, USA) as described [2, 45, 46]. Following transfer, the Immobilon membranes were further incubated with mSglt1-Ab (1:500) or actin-Ab (1:1000) at 4 °C overnight, extensively rinsed, and then incubated with corresponding secondary antibody, GAR-AP (0.1 μg/ml) or GAM-AP (0.5 μg/ml), at room temperature for 1 h. Where appropriate, mSglt1-Ab was blocked with the immunizing peptide by preincubation for 4 h at room temperature (final concentration of peptide 0.5 mg/ml) before use in immunoblotting assay. All other details related to handling of Immobilon membrane and visualization of protein bands with the BCIP/NBT-staining method were described in our previous publications [2, 46]. The labeled protein bands were scanned and densitometrically evaluated using the freely available software ImageJ (v.1.4) (http://imagej.nih.gov/ij/). The density of each protein band was expressed in arbitrary units, relative to the strongest band density (= 1) estimated in male mice. The amount of protein per well is indicated in the figure legends.

### Tissue fixation and immunocytochemistry

After sacrificing, various mouse organs/tissues were removed, rinsed with cold PBS, sliced, and fixed overnight in 4% *p*-formaldehyde. Tissue samples were then rinsed with four changes of a large amount of PBS (10–15 min each) to remove fixative and stored in PBS with 0.02% NaN_3_ at 4 °C until use. Further steps, which included tissue cryosectioning, an antigen retrieval protocol, and incubation with primary and secondary antibodies, were described in detail in our previous publications [2, 45, 46]. The optimal protocol to retrieve mSglt1 and Na^+^/K^+^-ATPase epitopes included a microwave oven heating in 10 mM citrate buffer, pH 6 [12]. Dilutions of primary antibodies were 1:25–1:300 for our non-commercial mSglt1-Ab, 1:100 for commercial anti-SGLT1 antibodies, and 1:100 for Na/K-ATPase-Ab. Dilutions of secondary antibodies were 1:800 for GAR-CY3 and 1:400 for GAM-FITC and DAG-CY3. In double-staining studies of mSglt1 and Na^+^/K^+^-ATPase, cryosections were first incubated with mSglt1-Ab (polyclonal antibody) at 4 °C overnight, followed by GAR-CY3 at room temperature for 1 h, then with Na/K-ATPase-Ab (monoclonal antibody) at 4 °C overnight, followed by GAM-FITC (1:100) at room temperature for 1 h. Where needed, mSglt1-Ab was preabsorbed with the immunizing peptide by incubation for 4 h at room temperature (final concentration of peptide 0.5 mg/ml) before its use in immunocytochemical assay. Furthermore, in either preliminary experiments (data not shown) or where necessary (shown in some figures), tissue cryosections or Western blot membranes were incubated only with the respective secondary antibodies (primary antibody was omitted), followed by usual steps in the staining procedure. The stained cryosections were covered with the fluorescence fading retardant Vectashield (Vector Laboratories, USA) and analyzed under fluorescence microscope OPTON III RS (Opton Feintechnik, Germany) using appropriate filters. The images were captured with the attached SPOT RT digital camera and software (Diagnostic Instruments, USA).

The pixel intensity of mSglt1-related CY3-fluorescence staining in the BBM of intestinal enterocytes and renal proximal tubules was measured using ImageJ (v.1.4). In intestine, one cryosection per segment (duodenum, jejunum, ileum) from each *mSglt1* ^*+/+*^ animal (three male and three female mice) was immunostained, and five to six random images were taken from each cryosection with a ×25 objective. In each image, six randomly chosen fluorescence-positive regions of interest (ROIs) in BBM were encircled; the pixel intensity was measured and subtracted for background fluorescence in non-stained basal parts of the same cells. The data, collected from one image, were averaged and used as one datum in further calculations. The results were expressed as relative (arbitrary) values to the strongest fluorescence pixel intensity measured in the BBM of jejunal enterocytes in male animals. In kidneys, one cryosection from each animal (four males and four females) was immunostained, and five randomly chosen images of the cortex/outer stripe were taken from each cryosection with a ×25 objective. In each image, five proximal tubule profiles (S2 in the cortex, S3 in the cortical medullary rays, S3 in the outer stripe) with the fluorescence-positive BBM were randomly chosen, the region of interest was encircled, and pixel intensity was measured. The pixel intensity of the staining in BBM of the same kind of tubule segments in each image was measured separately in cryosections from male and female kidneys, subtracted for background fluorescence in non-stained cells, averaged, and used as a single datum in further calculations. The results were expressed as relative (arbitrary) values to the strongest intensity measured in the BBM of proximal tubule S2 segments in male animals. The fluorescence intensity measurement was described in more detail previously [26, 30].

### Data presentation and statistical analysis

The Western blot and immunofluorescence images were imported in Adobe Photoshop 6.0 software for processing, assembling, and labeling. Numeric data (means ± SEM) were statistically analyzed using Student’s *t* test or ANOVA/Duncan test at the 5% level of significance.

## Results

### Demonstration of *mSglt1* mRNA in various organs in addition to intestine and kidney

Using specific primers in end-point RT-PCR analyses, in wild-type mice, the *mSglt1* mRNA transcripts were detected in the kidneys (Fig. S1), jejunum, liver, pancreas, eyes, tongue, lungs, salivary glands, prostate, and uterus (Fig. 1a). Under the same RT-PCR conditions, no specific transcripts were detected in testes, epididymides, adipose tissue, spleen, heart, skeletal muscle, cerebrum, and cerebellum. The mRNA expression of housekeeping gene *mRplp2* was similar in all the tested tissue samples (Fig. 1a). In *mSglt1* ^*−/−*^ mice, no *mSglt1*-related transcripts were observed neither in kidneys (Fig. S1) nor in all other tested organs and tissues (data not shown).

To compare the expression levels of *mSglt1* mRNA in various organs/tissues, qRT-PCR was performed. As shown in Fig. 1b, the *mSglt1* mRNA expression was very high in small intestine (duodenum). This is consistent with the observation that mSglt1 is the most abundantly expressed transporter in the BBM of mouse intestine [55]. The *mSglt1* mRNA abundance was relatively high in kidneys and salivary glands (parotid, submandibular, and sublingual); somewhat lower in prostate, tongue, eyes, and uterus; even lower in pancreas and lungs; quite low in liver; and very low in skeletal muscle, testes, spleen, heart, epididymides, adipose tissue, and brains (cerebrum and cerebellum). Except in kidneys (vide infra), the expression of *mSglt1* mRNA in other organs/tissues was similar in both sexes (data not shown). The mRNA expression level of the housekeeping gene *mHprt* was similar in all RNA samples (data not shown) and was thus used for normalization of qRT-PCR data.

### Expression of *mSglt1* mRNA and protein in gastrointestinal tract

For immunolocalization of mSglt1 protein, the previously described mSglt1-Ab was used [21]. Previously, we showed that this antibody, raised against a species-specific peptide of mSglt1, reacted with the luminal membrane of mouse jejunal enterocytes in *mSglt1* ^*+/+*^ mice but showed no reaction with enterocytes of *mSglt1* ^*−/−*^ mice. In the present study, we confirmed these observations by immunostaining the enterocyte BBM and by immunolabeling the broad ~ 85 kDa protein band in isolated small intestinal TCM (Fig. S2) and showed similar pattern in kidneys. Accordingly, mSglt1-Ab stained the luminal membrane of several cortical tubule profiles and labeled the ~ 75 kDa protein band in isolated renal TCM in wild-type mice, whereas these phenomena were missing in the kidney tissue samples from *mSglt1* ^*−/−*^ mice (Fig. S2). While different mobilities of the broad protein bands in small intestine and kidneys may reflect different glycosylation states and/or some other tissue-specific posttranslational modifications in enterocytes [39], species selectivity of mSglt1-Ab was demonstrated by showing absence of immunoreactivity in rat kidneys (data not shown).

To further determine the expression of mSglt1 along the gastrointestinal tract, we analyzed *mSglt1* mRNA by qRT-PCR and mSglt1 protein by immunostaining in stomach, duodenum, jejunum, ileum, cecum, and colon of wild-type mice (Fig. 2a). The highest concentration of *mSglt1* mRNA was detected in jejunum, followed by duodenum and ileum. The concentration of *mSglt1* mRNA in colon was low, less in cecum, and very low in stomach. Performing immunostaining with mSglt1-Ab, mSglt1 protein was demonstrated in the luminal membrane of duodenum, jejunum, and ileum (Fig. 2b) but could not be detected in stomach, cecum, and colon. The analysis of immunofluorescence intensity indicated a similar pattern of mSglt1 protein abundance in the BBM compared to *mSglt1* mRNA abundance, the highest staining being in jejunum, followed by duodenum and then ileum (Fig. 2b, c). No intestinal mSglt1-Ab-related staining was observed in *mSglt1* ^*−/−*^ mice (c.f. Fig. S2) and in *mSglt1* ^*+/+*^ mice after blocking the antibody with the antigenic peptide (data not shown).

### Distribution of mSglt1 in kidneys

We investigated the detailed nephron distribution of mSglt1 by immunocytochemistry. Immunostaining in kidneys from male *mSglt1* ^*+/+*^ mice is shown in Figs. 3 and 4. In the cortex, the BBM of proximal tubule S1 segment was unstained or very weakly stained (Figs. 3a inset and 4) whereas the BBM of S2 segment was strongly stained (Fig. 3). The staining intensity of the BBM in S3 segments was high in medullary rays and declined toward the outer stripe (Fig. 3a). In Fig. 3e, the staining intensity in the BBM of S2 and S3 segments in the medullary rays and outer stripe is compared.

In addition to the BBM of proximal tubules, the luminal membrane of TALH in the cortex and outer stripe and the luminal membrane of macula densa cells were stained (Figs. 3 and 4). In Fig. 4, the sections were double stained with Na/K-ATPase-Ab and mSglt1-Ab in order to determine localization of mSglt1 in the different nephron segments more clearly. As shown in Fig. 4a, Na/K-ATPase-Ab stained the basolateral invaginations of proximal tubule S1, S2, and S3 (in medullary rays) segments, and stronger the BLM of TALH and macula densa cells. mSglt1-Ab stained the luminal membrane of S2 and S3 segments, TALH, and macula densa, while the luminal membrane of S1 segments was largely negative (Fig. 4, images 1B and C) or occasionally very weakly positive (Fig. 4, images 2B and C). The merged images (Fig. 4c) confirm localization of mSglt1 protein in the luminal membrane of proximal tubule S2 and S3 segments, TALH, and macula densa. All localizations, stained with mSglt1-Ab in the kidneys of *mSglt1* ^*+/+*^ mice, remained unstained in *mSglt1* ^*−/−*^ mice (data not shown).

### Specific mSglt1-Ab-related immunoreactivity in salivary glands

Immunostaining with mSglt1-Ab was investigated in parotid, submandibular, and sublingual salivary glands of wild-type male and female mice (Fig. 5, *mSglt1* ^*+/+*^, red images and insets). In all three glands of both sexes, mSglt1 protein was detected in the luminal membrane of initial ducts (arrows). In parotid and sublingual glands, weak mSglt1-Ab immunoreactivity was also observed in the apical membranes of serous acinar cells (arrowheads in red images and insets). These structures remained unstained in the *mSglt1* ^*−/−*^ mice (Fig. 5). However, mSglt1-Ab immunoreactivity of some intracellular organelles in serous acinar cells of submandibular glands was not specific, being present also in *mSglt1* ^*−/−*^ mice.

### Immunolocalization of mSglt1 protein in tongue

In the tongue of *mSglt1* ^*+/+*^ mice, mSglt1-Ab-related immunostaining was strongest in the plasma membrane of epithelial cells located below and between the taste buds (Fig. 6a, arrows), while in *mSglt1* ^*−/−*^ mice, this staining was absent. To better define the cellular location of mSglt1 protein, we performed co-staining of mSglt1-Ab (Fig. 6b, red fluorescence) with Na/K-ATPase-Ab (Fig. 6b, green fluorescence). The merged picture in Fig. 6b shows that different to the Na^+^/K^+^-ATPase, which was mainly located in the mSglt1-negative basal cell layers (arrowheads), mSglt1-positive staining was observed in cells below and between the taste buds (arrows). In longitudinally sectioned taste buds, some apical mSglt1-Ab immunoreactivity suggests staining of the cilia of taste cells (double arrowheads in Fig. 6b).

### Immunolocalization of mSglt1 protein in pancreas

In the pancreas of *mSglt1* ^*+/+*^ mice, mSglt1 protein was immunolocalized to the luminal membrane of intralobular ducts (Fig. 7a, arrows), while in *mSglt1* ^*−/−*^, this staining was absent (Fig. 7b). To confirm this localization, we performed co-staining of mSglt1-Ab (red fluorescence, arrows) with Na^+^/K^+^-ATPase-Ab (green fluorescence, arrowheads) in wild-type (Fig. 7c, e) and *mSglt1* ^*−/−*^ mice (Fig. 7d, f). The merged pictures show the mSglt1 protein in the luminal membrane of intralobular (Fig. 7c) and interlobular (Fig. 7e) ducts (arrows) of *mSglt1* ^*+/+*^ mice but not in *mSglt1* ^*−/−*^ mice (Fig. 7d, f, arrows). No Sglt1-Ab immunoreactivity could be detected in the cells of Langerhans islets (Fig. 7e, LI)*.* Na^+^/K^+^-ATPase was positive in the plasma membrane of a variety of cells in wild-type and *mSglt1* ^*−/−*^ mice (Fig. 7c–f, green fluorescence, arrowheads).

### Immunolocalization of mSglt1 protein in liver

In cryosections of the liver tissue from wild-type mice, which were double stained with mSglt1-Ab and Na^+^/K^+^-ATPase-Ab, the red-stained mSglt1 protein was exclusively located in the luminal domain of bile ducts in portal area (Fig. 7, LIVER, *mSglt1* ^*+/+*^, mSglt1 and Merge, arrows), whereas the green-stained Na^+^/K^+^-ATPase was located in the sinusoidal membrane of hepatocytes (arrowheads) and in the BLM of bile ducts (arrows) (Fig. 7, LIVER, Na/K-ATPase, and Merge). The mSglt1-Ab-related staining in bile ducts was not observed in *mSglt1* ^*−/−*^ mice (Fig. 7, LIVER, *mSglt1* ^*−/−*^).

### Immunolocalization of mSglt1 protein in prostate and periurethral glands

In the prostatic gland, mSglt1-Ab immunoreactivity was observed in the luminal membrane of follicular epithelial cells (Fig. 8a, Ab, arrows) and in myoepithelial cells around the follicles (Fig. 8a, Ab arrowheads). Staining in both locations was abolished after preincubation of the antibody with antigenic peptide; however, only the staining in epithelial cells proved to be specific because only staining in this location was absent in *mSglt1* ^*−/−*^ mice (Fig. 8a, Ab + peptide).

In periurethral glands of wild-type mice (Fig. 8b), mSglt1-Ab stained the luminal membrane of acinar cells and excretory ducts (8B, Ab arrows). This staining was specific because it was neither observed after preincubation of the antibody with antigenic peptide nor in *mSglt1* ^*−/−*^ mice. In addition, strong staining of some intracellular, densely packed granular material was observed which disappeared after preincubation of the antibody with antigenic peptide; however, it proved to be non-specific because it was also observed in *mSglt1* ^*−/−*^ mice (8B, Ab, arrowheads).

### Immunolocalization of mSglt1 protein in eyes

In the eyes of *mSglt1* ^*+/+*^ mice, mSglt1-Ab stained the axons of retinal ganglion cells and neurons in the optic nerve (Fig. 9a, Ab, arrows), the optic nerve central artery (Fig. 9a, Ab, arrowhead), and blood vessels in the retina (data not shown) and its periphery (Fig. 9b, Ab, arrowheads). Staining of the axons and ganglion cells was specific because it was not observed after preincubation of the antibody with antigenic peptide and in *mSglt1* ^*−/−*^ mice. In contrast, the staining of the blood vessels proved to be non-specific, being present also in *mSglt1* ^*−/−*^ mice and absent with the peptide-blocked antibody.

### Demonstration of divergent sex dependence of Sglt1 expression in kidneys between mouse and rat

The above-described expression studies were performed in tissue samples from male and female mice. Except in kidneys, no clear sex differences in either *mSglt1* mRNA and/or protein expression were observed. Previously, we demonstrated that the expression of Sglt1 in rat kidneys and the expression of Sglt2 in rat and mouse kidneys are sex dependent, and that the sex-dependent Sglt2 expression differs between mice and rats [2, 45, 46]. In this study, we analyzed the *mSglt1* mRNA expression by qRT-PCR, and the protein expression by immunocytochemistry in tissue cryosections and by Western blotting of BBM isolated from whole kidneys of wild-type mice. As shown in Fig. 10a, the expression of *mSglt1* mRNA was ~ 60% higher in females than in males. In contrast, the mSglt1-Ab-related staining intensity in the BBM of cortical and outer stripe tubules was visibly stronger in males (Fig. 10b). Staining intensity at the BBM of proximal tubule S2 segments in the cortex, and of S3 segments in the outer stripe was ~ 20 and ~ 130% higher in males than in females, respectively (Fig. 10c). Sex differences in protein expression were confirmed by Western blotting of isolated TCM and BBM. The density of mSglt1-related ~ 75 kDa protein band was ~ 60% (TCM) and ~ 33% (BBM) stronger in males compared to females, whereas the density of actin band associated with BBM was sex-independent (Fig. 10d, e). The dissociation between mRNA and protein abundance was not observed in rats, where both *rSglt1* mRNA and protein were female dominant [2].

### Side to side comparison of *Sglt1* mRNA abundance in various organs/tissues from mice and rats

Comparing relative abundance of *Sglt1* mRNA between organs of adult male rats and mice species differences were detected. We determined the abundance of *Sglt1* mRNAs in kidneys, pancreas, lungs, liver, skeletal muscle, heart, cerebrum, and cerebellum. As shown in Table 2, in the mouse pancreas, liver, and skeletal muscle, the relative *mSglt1* mRNA expression was 4-, 2-, and 11-fold higher, respectively, than in the same rat organs. On the other side, in lungs, the relative expression was 8-fold higher in rat compared to mouse, while the relative expression in heart, cerebrum, and cerebellum was similarly low in both species.

**Table 2:** Comparison of *Sglt1* mRNA expression in selected mouse and rat organs

Organ/tissue	Mouse	Rat
Kidneys	100 (± 18.05)	100 (± 7.38)
Pancreas	10.95 ± 0.15	2.69 ± 0.36*
Lungs	7.96 ± 0.72	63.89 ± 9.31*
Liver	2.17 ± 0.31	1.02 ± 0.12*
Skeletal muscle	0.99 ± 0.18	0.09 ± 0.01*
Heart	0.09 ± 0.04	0.11 ± 0.07
Cerebrum	0.04 ± 0.00	0.03 ± 0.009
Cerebellum	0.03 ± 0.01	0.01 ± 0.003

Relative expression of *mSglt1* and *rSglt1* mRNA was analyzed by comparative 2^−ddCt^ method. The data were normalized to the housekeeping genes *mHprt* and *rHprt*, whose expression was similar in the tested organs of mice and rats, respectively (data not shown). Results are presented as percentage of the *Sglt1* mRNA expression in kidneys. qRT-PCR data (mean ± SEM) were obtained with cDNA samples of independent RNA preparations from three males of each species

*P < 0.02 vs. mouse

### Absence of specific mSglt1-Ab immunoreactivity in the organs/tissues with low expression of *mSglt1* mRNA

Specific immunoreactivity with mSglt1-Ab could not be detected with different antigen unmasking techniques in lungs, heart, brain, testes, ductuli efferentes, epididymides, spleen, skeletal muscle, and adipose tissue. These organs exhibited low to very low expression of *mSglt1* mRNA (c.f. the data in Fig. 1). Whereas in some organs, such as testes, spleen, skeletal muscle, and adipose tissue, no mSglt1-Ab-related immunoreactivity was observed (data not shown), in few organs such as lung, heart, and brain, the same immunoreactivities were observed in *Sglt1* ^+/+^ and *Sglt1* ^−/−^ mice. This non-specific immunoreactions were abolished when the antibody was preabsorbed with the antigenic peptide. The data from lung, heart, and brain are presented in Figs. S3, S4, and S5.

In the lung of wild-type and *mSglt1* knockout mice, non-specific peptide-blocked mSglt1-Ab immunoreactivity was observed in peribronchial myoepithelial cells and the wall of arteries (Fig. S3). In heart ventricles of some *mSglt1* ^*+/+*^ and some *mSglt1* ^*−/−*^ mice, mSglt1-Ab stained arterial walls with variable intensity (Fig. S4). Using peptide-blocked mSglt1-Ab, no staining of arterial walls was observed. Previously, we described immunostaining of cerebral neurons in brain of rat, rabbit, and pig with antibodies directed against SGLT1 peptides [41, 45]. Being abolished when the antibody had been preabsorbed with respective antigenic peptides, this staining was considered specific. Here, we performed immunostaining of cryosections from cerebrum and cerebellum with mSglt1-Ab using preabsorption of the antibody with antigenic peptide and *mSglt1* ^*−/−*^ mice as specificity controls. Immunostaining in cryosections of the cerebrum is summarized in Fig. S5. In *mSglt1* ^*+/+*^ mice, mSglt1-Ab stained the pia mater and arterial wall (Fig. S5A, Ab, arrows and arrowhead, respectively), pyramid cells in the cortex (Fig. S5B, Ab, arrows), bunches of neurons in the medulla (Fig. S5C, Ab, arrows), and epithelial cells of the choroid plexus (Fig. S5D, Ab, arrows). Noteworthy, the same mSglt1-Ab immunoreactivity was observed in *mSglt1* ^*−/−*^ mice. The staining in both wild-type and knockout mice was similar in male and female brains and was abolished when mSglt1-Ab was preabsorbed with the antigenic peptide (Ab + peptide). Similar results were obtained in cerebellum (data not shown).

### Characterization of immunocytochemical reactions of three commercial antibodies against SGLT1/Sglt1 in kidney, small intestine, lung, heart, and brain

Wondering why the specific location of mSglt1 protein could not be detected in lungs, heart, and brains with our mSglt1-Ab, we performed additional immunocytochemical experiments with tissue cryosections by employing three commercial anti-SGLT1 antibodies, no. 07-1417 from Merck Millipore (Merck Millipore-Ab), ab-14686 from Abcam (Abcam-Ab), and sc-20582 from Santa Cruz (Santa Cruz-Ab). These antibodies have been used by other scientists for various immunochemical studies in rodents [1, 3, 4, 9, 28, 40, 42, 51, 56, 58]. To test their specificity, we first performed immunocytochemistry in cryosections of renal cortex and outer stripe and of small intestine (duodenum) in *mSglt1* ^*+/+*^ and *mSglt1* ^*−/−*^ mice (Figs. S6, S7, and S8).

With Merck Millipore-Ab, similar immunoreactions were observed in *mSglt1* ^*+/+*^ and *mSglt1* ^*−/−*^ mice showing overlap with the distribution of mSglt1 in both, kidney and duodenal tissue (Fig. S6). This indicates that this antibody is not at all specific for mSglt1. With Abcam-Ab and Santa Cruz-Ab, distinct staining of the BBM in small intestinal enterocytes was observed that was absent in *mSglt1* ^*−/−*^ mice suggesting selectivity in the intestine (Figs. S7 and S8). However, staining for mSglt1 in the kidney with these antibodies revealed unsatisfactory results; staining of luminal domain of proximal tubular segments was relatively weak in *mSglt1* ^*+/+*^ mice and was not completely absent in *mSglt1* ^*−/−*^ mice. In addition, these antibodies stained the cells in collecting ducts in both *mSglt1* ^*+/+*^ and *mSglt1* ^*−/−*^ mice (Figs. S7 and S8).

As shown in Figs. S9, S10, and S11, performing parallel staining of cryosections from the lung, heart, and brains (cerebrum and cerebellum) of *mSglt1* ^*+/+*^ and *mSglt1* ^*−/−*^ mice with Merck Millipore-Ab, Abcam-Ab, and Santa Cruz-Ab, respectively, no specific localization of mSglt1 protein could be detected although different antibody concentrations were used and different antigen unmasking techniques were applied (data not shown). With Merck Millipore-Ab in lungs of both wild-type and Sglt1 knockout mice, a relatively strong immunoreactivity was observed in bronchial epithelum, peribronchial myoepithelial cells, and arterial walls, whereas Abcam-Ab and Santa Cruz-Ab only exhibited non-selective background staining which was also observed with the secondary antibody (Fig. S9). In the heart (Fig. S10), Merck Millipore-Ab strongly stained the surface of cardiomyocytes; however, this staining was observed in both wild-type and knockout mice (Fig. S10). The two other antibodies only showed background staining. In the brain (cerebrum) of both *mSglt1* ^*+/+*^ and *mSglt1* ^*−/−*^ mice, bunches of neurons were stained with all three commercial antibodies (Fig. S11).

Taken together, Merck Millipore-Ab is not suitable for immunocytochemical investigation of Sglt1 in mouse. Abcam-Ab and Santa Cruz-Ab show specificity for immunocytochemical localization of mSglt1 in mouse small intestine; however, they exhibit non-specific reactions in other organs.

## Discussion

Performing immunocytochemistry in mice and using *Sglt1* ^*−/−*^ mice as negative control, new insights concerning limitations of previously performed immunocytochemical localizations of SGLT1/Sglt1 in various tissues of different species are provided. First, we showed that preabsorption of antibodies with their antigenic peptides is not sufficient for an unambiguous verification of specific antibody reactions in immunocytochemistry. Second, we observed functionally relevant species differences between the expression of SGLT1 in rodents and humans. Third, also significant differences in expression and location of Sglt1 in mice and rats were identified. Finally, previously described immunolocalizations of Sglt1 in mice were re-evaluated and several new locations of Sglt1 were identified (see Table 3 for overview). An evaluation of three commercial antibodies used for immunolocalizations of Sglt1 in mouse tissues revealed their insufficient specificities.

**Table 3:** Overview of the determined expression pattern of Sglt1 in mouse organs/tissues at mRNA and protein levels

	mRNA	Protein	Localization
Gastrointestinal tract			
Stomach	+*	ND	–
Small intestine	++++	+	Enterocytes, BBM
Cecum	+*	ND	–
Colon	+*	ND	–
Kidneys	+++	+	Proximal tubule S2/S3, BBM
			TALH in cortex and outer stripe, LM
			Macula densa, LM
Pancreas	+	+	Pancreatic ducts (intra-/interlobular), LM
Liver	+	+	Bile ducts, LM
Spleen	+*	ND	–
Lungs	+	ND	–
Heart	+*	ND	–
Skeletal muscle	+*	ND	–
Adipose tissue	+*	ND	–
Brain			
Cerebrum	+*	ND	–
Cerebellum	+*	ND	–
Eyes	++	+	Axons of ganglion cells, optic nerve
Tongue	++	+	Epithelial cells surrounding taste buds, PM
Salivary glands			
Parotid	+++	+	Serous acinar cells and initial ducts, LM
Submandibular	+++	+	Initial ducts, LM
Sublingual	+++	+	Serous acinar cells and initial ducts, LM
Male reproductive tract			
Testes	+*	ND	–
Epididymides	+*	ND	–
Prostate	++	+	Epithelial cells in follicles, LM
Periurethral glands	NT	+	Acinar cells and excretory ducts, LM
Female reproductive tract			
Uterus^a^	++	+	Endometrial epithelium, LM

Expression of *mSglt1* mRNA and mSglt1 protein localization was determined by qRT-PCR and immunofluorescence studies. Expression of *mSglt1* mRNA in the tissue was very high (++++), high (+++), medium (++), low (+), or very low (+*). Positive findings of mSglt1 protein by immunostaining in the organs/tissues of wild-type mice (negative in *Sglt1* knockout mice) are indicated with (+), whereas no detection is indicated with (ND). BBM brush-border membrane, LM luminal membrane, PM plasma membrane. ^a^The immunocytochemical data will be shown elsewhere (MD Salker, Y Singh, N Zeng, H Chen, S Zhang, AT Umbach, H Fakhri, U Kohlhofer, L Quintanilla-Martinez, RRP Durairaj, FSV Barros, P Vrljicak, S Ott, S Brucker, D Wallwiener, I Vrhovac Madunić, D Breljak, I Sabolić, H Koepsell, JJ Bronsen and F Lang; Scientific Reports, Accepted for publication)

Studying the expression of Sglt1 in different organs and tissues of mice, we utilized the possibility to employ samples from *mSglt1* ^*−/−*^ mice as negative controls [21]. We validated the *mSglt1* ^*−/−*^ control by showing that neither mRNA in all tested organs and tissues nor mSglt1 protein in small intestine and kidney were detected in *mSglt1* ^*−/−*^ mice (Figs. S1 and S2). Comparing the knockout mouse control with preabsorption of our custom-made mSglt1-Ab with antigenic peptide, we often obtained the expected results, particularly in organs with high expression of Sglt1 such as intestine and kidney. However, in some organs, such as submandibular glands, lung, heart, brain, prostate, periurethral glands, and eyes, mSglt1-Ab immunoreactivity in various locations disappeared when mSglt1-Ab had been absorbed with the antigenic peptide but not in the *mSglt1* ^*−/−*^ mouse. Thus, employing the *mSglt1* ^*−/−*^ mouse as control, mSglt1-Ab immunoreactions with mSglt1 protein and proteins containing a cross-reacting epitope could be distinguished. The importance of a reliable specificity control in addition to the quality of the employed antibodies is emphasized by our re-evaluation of the specificity of three commercial antibodies using the *mSglt1* ^*−/−*^ as control. However, although the information in the respective technical sheets indicate their possible reactivity with the human and rodent SGLT1/Sglt1 proteins, here, we showed that one commercial antibody (no. 07-1417 from Merck Millipore) reacts with a protein with similar distribution as mSglt1, but apparently not with mSglt1. The two other antibodies (no. 14686 from Abcam and sc-20582 from Santa Cruz) reacted weakly with Sglt1 protein, but they also exhibited non-specific reactions in the kidney, which did not show up with our custom-made mSglt1-Ab. These two commercial antibodies showed Sglt1-specific immunoreactivity in mouse small intestine where mSglt1 is most abundantly expressed; however, they exhibited non-specific cross-reactions in the lung, heart, and brain. Thus, they are not appropriate to determine locations of Sglt1 in these and others tissues with low expression of Sglt1. Our immunocytochemical study with the three commercial antibodies do not exclude that these antibodies may show more specificity in Western blots with mouse tissues; however, this has to be verified using *mSglt1* knockout controls.

Considering the limited validity of the preabsorption control for proof of antibody specificity, the previously determined locations of SGLT1/Sglt1 in different organs and tissues must be furnished with a question mark unless the same immunocytochemical location has been obtained with antibodies against different epitopes and/or independent evidence for the specific location has been provided such as in situ hybridization or functional studies ([31, 54] and references in there). The situation becomes puzzling in organs with low SGLT1/Sglt1 expression indicated by low expression of *SGLT1/Sglt1* mRNA, when the available functional tests are not selective for SGLT1 and/or a specific cellular location of SGLT1 as in the case of Sglt1 in the brain of rodents ([31, 59] and references in there).

Between humans on one hand and rats and mice on the other, similarities and distinct differences were observed. For example, in humans and these rodents, the highest *SGLT1/Sglt1* mRNA concentrations were observed in small intestine, followed by the kidney. At variance in human heart, where a high concentration of *SGLT1* mRNA was measured [3, 4, 14, 39, 61], in hearts of rats and mice, low concentrations of *Sglt1* mRNA were determined (Table 2 and [2, 45, 54]). In some organs, the expression of *Sglt1* mRNA was also significantly different between mice and rats. For example, the *mSglt1* mRNA concentrations in pancreas and skeletal muscle were 4- and 11-fold higher, respectively, in mice compared to rats. At variance, the *mSglt1* mRNA concentration in lung was 8-fold lower in mice compared to rats (Table 2). Consistently, we observed mSglt1-Ab immunoreactivity in mouse pancreas but not in mouse lung (Figs. 7 and S3). Species differences were also observed in the immunolocalization of SGLT1/Sglt1 protein. For example, different to SGLT1 in humans, in rats and mice, Sglt1 was localized to the TALH and macula densa (Figs. 3 and 4) [2, 54].

In the present study, we show that the location of mSglt1 protein in mouse small intestine and liver, as observed with mSglt1-Ab using the *Sglt1* ^*−/−*^ mouse as controls, is similar to the locations described in rats and humans using preabsorption with antigenic peptides as controls [2, 54]. This indicates the absence of large species difference in these organs and strongly suggests the validity of our previous data in rats and humans. In the mouse, rat, and human small intestine, Sglt1/SGLT1 was localized in the BBM of enterocytes showing the highest concentration in jejunum followed by duodenum and ileum indicating that bulk glucose absorption takes place in these segments (this study, and [2, 21, 50, 54]). This pattern of protein abundance in the intestinal segments is consistent with the pattern of mRNA expression, which indicated the small intestine as the site of highest expression of SGLT1/Sglt1 mRNA among mammalian organs (this study, and [14, 31, 61] and reference in there). In addition, in the human [54], mouse [21], and rat (D. Kleesen, I. Sabolic and H. Koepsell, unpublished data) small intestine, SGLT1/Sglt1 protein was also localized in L-cells and K-cells. Thus, mice and rats can be considered as valid models for SGLT1 localization and SGLT1-mediated functions in the human small intestine. The expression of Sglt1/SGLT1 in stomach, cecum, and colon appears to be very low (this study, and [2, 14, 54, 61]).

The expression of *mSglt1* mRNA in liver, as shown in this study in mouse, was previously detected in human [14]. Similar to the observations in rats [2] and humans [54], also in mice, mSglt1 protein was immunolocalized to the luminal membrane of bile ducts. In bile ducts, SGLT1 may play a key role in regulating bile formation in mammals. As shown in rats, glucose enters the bile passively and is thereafter reabsorbed actively in bile ducts [16, 23], obviously via Sglt1 in the duct epithelial cells. Glucose can exit the bile duct cell across the BLM by facilitated diffusion via GLUT1 [32]. Glucose uptake absorption in the bile ducts generates an osmotic driving force for water absorption via water channels in the apical and basolateral membranes [35, 36, 48].

It is generally accepted that SGLT2 in the early proximal tubule and SGLT1 in the late proximal tubule are critically involved in the reabsorption of glucose from the renal ultrafiltrate [27, 31]. Previous immunolocalizations of Sglt1/SGLT1 in rat and human ([2, 54] and references in there), and the immunolocalization of mSglt1 in mouse described in this manuscript, are consistent with this concept; however, some functional relevant species differences became apparent. Similar to human and rat, also in mouse, mSglt1 protein was largely localized to the BBM of proximal tubules and showed the highest staining intensity in the more distal parts following the order: cortical S2 segment > S3 segment in medullary rays > S3 segment in outer stripe >>> cortical S1 segment. For the immunostaining of rSglt1 in rat, a different order of staining intensities was observed: S3 segment in outer stripe > S3 segment in medullary rays > cortical S2 segment > cortical S1 segment [2, 33, 49]. In contrast, in the human nephrons, immunoreactivity of SGLT1 was only detected in S3 segments, showing a higher staining intensity in the S3 segment of the outer stripe compared to the S3 segments in medullary rays [54]. Different to human, in rat [2] and mouse (Figs. 3 and 4), Sglt1 proteins were also immunolocalized to the luminal membrane of cortical TALH including the macula densa. The TALH in the outer stripe was stained in mice but not in rats. The observation that TALH in the inner stripe were neither stained in mice nor in rats suggests that only the short loops of Henle are involved in Sglt1-mediated glucose reabsorption. This is consistent with the data showing phlorizin-inhibitable Na^+^-d-glucose cotransport and net sodium and water reabsorption in short loops of Henle in rats [7]. The functional role of Sglt1 in the macula densa of rats and mice is not understood. We speculate that an increased glucose concentration in distal tubules may participate in glomerulotubular feedback by increasing intracellular sodium via Na^+^-d-glucose cotransport. Assuming functional relevance of Sglt1 in the macula densa for tubuloglomerular feedback, different effects of Sglt1 inhibitors in these animal models compared to humans are anticipated. For example, the decrease of glomerular filtration, observed during the treatment of diabetic mice with the Sglt2 inhibitor empagliflozin [20, 52], may be due to increased Sglt1-mediated Na^+^-d-glucose cotransport in the macula densa. This effect should not be relevant in humans.

Performing preclinical studies with inhibitors of SGLT2, SGLT1, or SGLT2 plus SGLT1 using rats or mice, sex differences in the renal expression of SGLT1 have to be considered. Whereas no sex-dependent expression of SGLT1 protein has been detected in human kidneys [54], a different sex dependence of the renal expression of *Sglt1* mRNA and Sglt1 protein has been observed between rat [2] and mouse (Fig. 5). In the kidneys of mice and rats, higher expressions of *Sglt1* mRNA were observed in females compared to males. However, in mouse kidneys, higher amounts of Sglt1 protein were found in males compared to females, whereas in rat kidneys, higher abundance of Sglt1 protein was observed in females. The dissociation between mRNA and protein abundance of Sglt1, suggesting differences in message stability or regulation of protein synthesis, and/or protein turnover, has also been observed for Sglt2 in rats and mice, and after inhibition of Sglt2 by empagliflozin [46, 52, 53].

In the present study, we re-evaluated the immunolocalization of mSglt1 in several mouse organs and demonstrated several novel locations of SGLT1. In the tongue, we detected specific mSglt1-Ab immunoreactivity strongly in the cells around and below gustatory buds whereas we only observed very weak staining of cells within taste buds and some staining at the tips of the buds which may indicate staining of taste cilia. These data are at variance with the previously reported immunolocalization of Sglt1 protein in a subset of cells of mouse gustatory buds [51, 57]. Since the previous immunolocalization in taste buds was performed with the antibody ab14686 from Abcam using the omission of the primary antibody as specificity control, the previous localization may represent an artifact. Both Sglt1 in cells within taste buds and Sglt1 in cells surrounding taste buds may be involved in sweet sensing ([13] and references in there).

As previously reported in the rats [28, 29, 43], we observed *mSglt1* mRNA expression in salivary glands of mice (Fig. 1). By immunostaining of salivary glands, mSglt1 protein was localized to the luminal membranes of initial segments of the salivary ducts, as has been observed in rat [2, 43]. In mouse, we observed in addition specific mSglt1-Ab immunoreactivity in the luminal membrane of parotic and sublingual acinar cells (Fig. 5). In salivary glands, SGLT1 may be critically involved in reabsorption of glucose from iso-osmotically secreted primary saliva. In humans, the measured d-glucose concentration in unstimulated mixed saliva was 15–300-fold lower than in plasma [22]. Glucose absorption may create a transmembrane osmotic gradient that induces water reabsorption via water channels contributing to the concentration of saliva [17, 38]. In pancreas, where we confirmed a relatively low expression of *mSglt1* mRNA reported before in rat and human organs [9, 61], and localized mSglt1 protein to the luminal membranes of intra- and interlobular ducts, mSglt1 may have a similar function. However, with our custom-made mSglt1-Ab, we could not confirm the localization of Sglt1 protein in alpha cells of Langerhans islets, which was recently reported by Bonner et al. in human pancreas [9] using the antibody no. 07-1417 from Merck Millipore.

In the prostate, we demonstrated specific location of mSglt1 protein in the luminal membrane of follicular epithelium, which was suggested from earlier, less well-controlled immunocytochemical data on SGLT1 obtained in diseased human prostate [8, 47].

Novel specific localizations of mSglt1, observed in this study, were the apical membrane of secretory cells and the luminal membrane of ducts in periurethral glands, axons of retinal ganglial cells, and the optic nerve. A resolution of the immunostaining does not allow us to distinguish whether mSglt1 is located in nerve fiber plasma membranes or enwrapping myelin-forming oligodendrocytes. If the locations in the eye are also present in humans, they may be of biomedical relevance, and SGLT1 inhibitors have to be tested for possible side effects in the eye.

The expression and potential biomedical importance of SGLT1/Sglt1 in the brain has been a matter of numerous studies and disputes ([31] and references therein). The observation made in the present study that mSglt1-Ab immunostaining of brain neurons and ependymal cells could be blocked with antigenic peptide, but was also observed in *mSglt1−/−* mice, suggesting the absence or minor abundance of mSglt1 protein in mouse brain in the normal functional state. The data raise doubts whether our previous immunocytochemical location of Sglt1 in neurons of rat brain using an antibody against an equivalent epitope of rat Sglt1 and preabsorption for specificity control [2] is valid. The possibilities that proteins which cross react with an antibody against SGLT1/Sglt1 are located at identical positions as SGLT1/Sglt1, and that in case of low abundance SGLT1/Sglt1 expression no immunoreaction over background is observed, do not prove that SGLT1/Sglt1 is absent. This may be due to the limitation of the sensitivity of the antibody to detect small amounts of the transporter. As discussed previously, the expression of Sglt1 in neurons and small vessels of rat brain has been suggested by functional studies ([31] and references in there). For example, in rat brain, uptake of SGLT/Sglt-specific radioactive substrates into different brain regions and into an experimentally induced epileptic focus has been demonstrated [41, 59].

The failure to detect a specific immunoreaction in mouse lung with our antibody against mSglt1 was surprising because the amount of *mSglt1* mRNA is 3.7-times higher compared to liver where we observed specific mSglt1 immunoreactivity. This may be partially due to different correlations between mRNA and protein abundance in lung and liver and/or to tissue-specific differences in background that masks specific immunoreactivity. The high abundance of *SGLT1* mRNA in human and rat lungs suggests that the previously described immunocytochemical location of SGLT1/Sglt1 in type 2 alveolar cells and Clara cells in human and rat lungs [54] is valid. Because 8-fold higher amounts of *Sglt1* mRNA were detected in the lung of rat versus mouse, and glucose-dependent sodium reabsorption from rat bronchi has been observed [5], the expression of Sglt1 protein in rat is probably similar to humans.

The failure to detect specific immunoreactivity with our antibody in mouse heart in contrast to human heart is not surprising considering very low abundance of *Sglt1* mRNA in mouse heart compared to the abundant *SGLT1* mRNA in human heart [14, 54, 61]. Although the immunolocalization of Sglt1 in sarcolemma of mouse heart [4] may be an artifact, because it was performed with antibody sc-20582 from Santa Cruz which proved to be not specific for immunolocalization of Sglt1 in mouse, expression of Sglt1 protein in mouse heart cannot be excluded. Data showing that the knockdown of Sglt1 in heart attenuated cardiomyopathy that was induced by activation of AMP-activated protein kinase [42] suggest that some Sglt1 protein is expressed.

In conclusion, our study provided new insight in the location of Sglt1 in several organs of mice. Some unexpected differences between the expression, location, and sex dependence of Sglt1 in the mouse and rat organs, which are also different from those in humans, were detected indicating the presence of functional relevant species differences. Various extrarenal localizations of SGLT1 represent possible health risk in case of using novel inhibitors for diabetes treatment in humans, which are primarily aimed to inhibit the SGLT1-mediated reabsorption of glucose in kidneys ([31, 60] and references in there). Species differences between mice, rats, and humans in the expression of this transporter in various organs strongly confine the use of mice as models for the elucidation of side effects of SGLT1 inhibitors in humans. Finally, our study proved the animal knockout model as the only relevant test of the antibody specificity in immunochemical studies.

## Electronic supplementary material

ESM 1 (PDF 2275 kb)
