# Supplementary material for: Expression profiling and immunolocalization of Na+-d-glucose-cotransporter 1 in mice employing knockout mice as specificity control indicate novel locations and differences between mice and rats
Source: Pflugers Arch. 2017 Aug 26;469(12):1545–65. doi: 10.1007/s00424-017-2056-1 (PMC5691098; doi:10.1007/s00424-017-2056-1)
Supplement: Supplementary file 1 — (PDF 2275 kb) [file 424_2017_2056_MOESM1_ESM.pdf]

SUPPLEMENTARY MATERIAL

Expression profiling and immunolocalization of Na^+-D-glucose-cotransporter 1 in mice employing knockout mice as specificity control indicate novel locations and differences between mice and rats

Ivana Vrhovac Madunić, Davorka Breljak, Dean Karaica,

Hermann Koepsell and Ivan Sabolić

Molecular Toxicology Unit, Institute for Medical Research and Occupational Health, Zagreb, Croatia, and Department of Molecular Plant Physiology and Biophysics, University of Würzburg, Würzburg, Germany

Evaluation of wild type (mSglt1$^{+/+}$) and knockout (mSglt1$^{-/-}$) mice by mRNA expression in kidney tissue

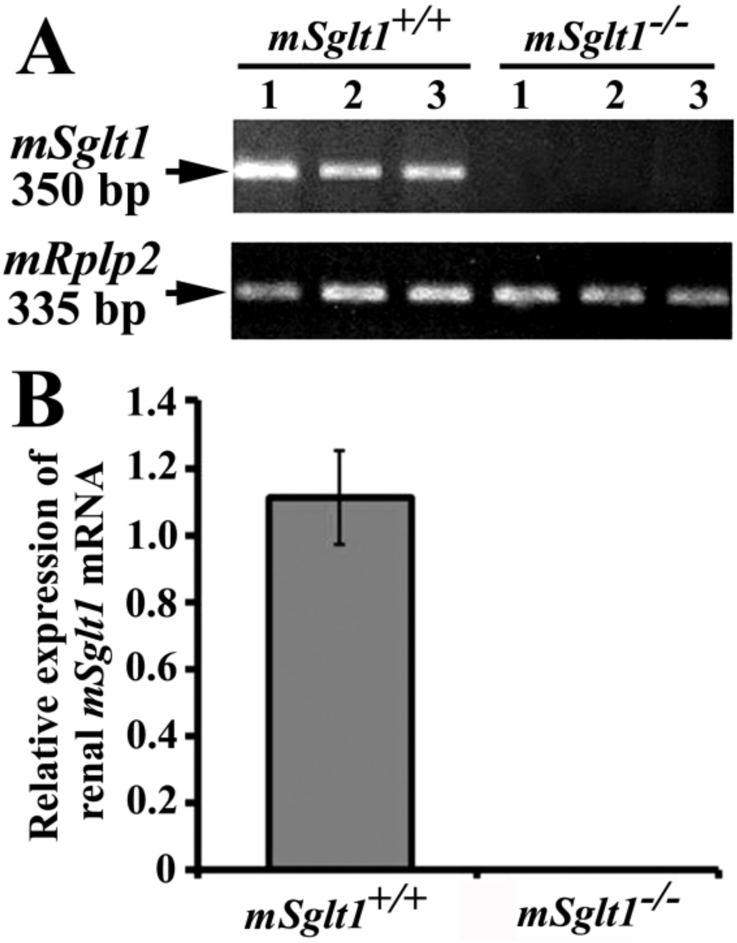

Fig. S1. Demonstration that *mSglt1* mRNA is not expressed in kidneys of *mSglt1*$^{-/-}$ mice. **A.** End-point RT-PCR; the *mSglt1*-related PCR product of 350 bp observed in wild type (*mSglt1*$^{+/+}$) mice was absent in knockout (*mSglt1*$^{-/-}$) mice. The mRNA expression of housekeeping gene *mRplp2* (PCR product of 335 bp) was positive in both *mSglt1*$^{+/+}$ and *mSglt1*$^{-/-}$ mice. The bands represent mRNA expression in tissue samples prepared from 3 male animals of each genotype. **B.** Quantitative RT-PCR in kidney of *mSglt1*$^{+/+}$ and *mSglt1*$^{-/-}$ mice; *mSglt1* mRNA was detected in kidneys of *mSglt1*$^{+/+}$ but not in *mSglt1*$^{-/-}$ mice. Means ± SEM from 3 male mice of each genotype are shown. The methods are described in the main paper.

Evaluation of mSglt1-Ab by immunochemical methods in mSglt1$^{+/+}$ and mSglt1$^{-/-}$ mice

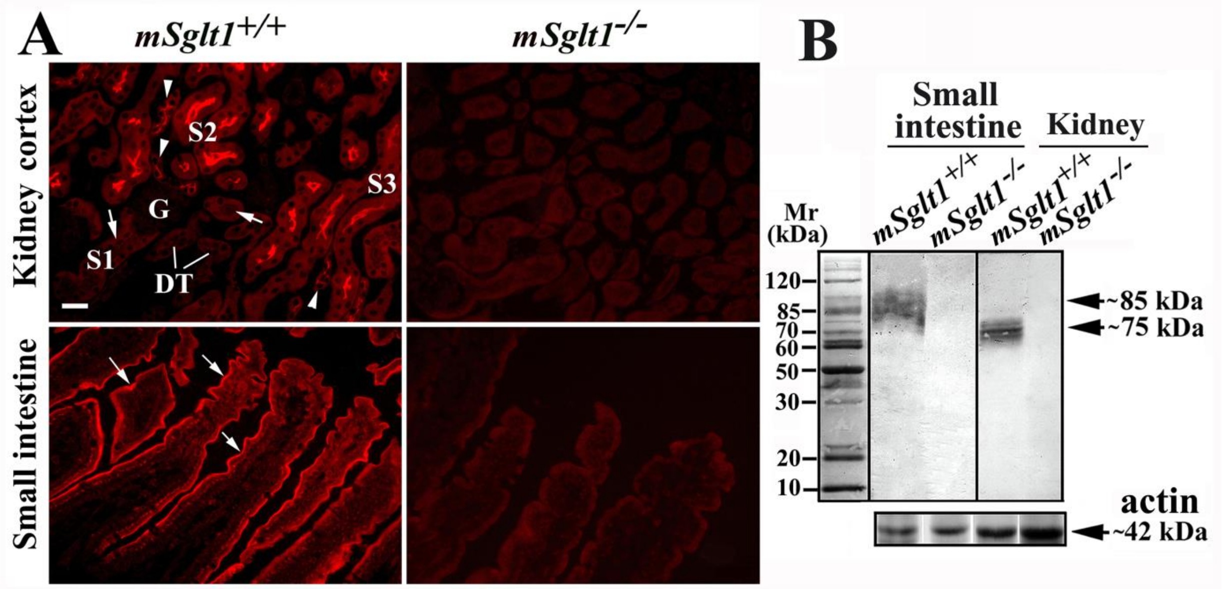

Fig. S2. Evaluation of the specificity of mSglt1-Ab immunoreactivity in kidney cortex and small intestine (jejunum) employing *mSglt1*$^{+/+}$ and *mSglt1*$^{-/-}$ mice. Immunocytochemistry was performed in cryosections (A) and immunolabeling in Western blots of isolated total cell membranes (TCM) (B). The methods are described in the main paper. **A.** In kidney cortex of *mSglt1*$^{+/+}$ mice, mSglt1-Ab stained the luminal domain of proximal tubular S2 and S3 segments, and of the thick ascending limb of Henle loop (arrowheads). Glomeruli (G), S1 segments (arrows), and convoluted distal tubules (DT) remained unstained. In small intestine (jejunum), the brush-border of enterocytes was strongly stained (arrows). In *mSglt1*$^{-/-}$ mice, the immunoreactions in both kidney and small intestine were absent. Bar, 20 µm for all images. **B.** In TCM from the small intestine and kidneys of *mSglt1*$^{+/+}$ mice, mSglt1-Ab labeled a single protein band of ~85 kDa and ~75 kDa, respectively. The bands were absent in TCM from *mSglt1*$^{-/-}$ mice. The ~42 kDa protein band of actin, used as a loading control, was positive in both *mSglt1*$^{+/+}$ and *mSglt1*$^{-/-}$ mice. 40 µg of protein were applied per lane. The data are representative for similar findings in 3 male mice of each genotype.

Mouse organs in which specific immunoreactivity of mSglt1-Ab was not detected (Fig. S3 lung, Fig. S4 heart, Fig. S5 brain)

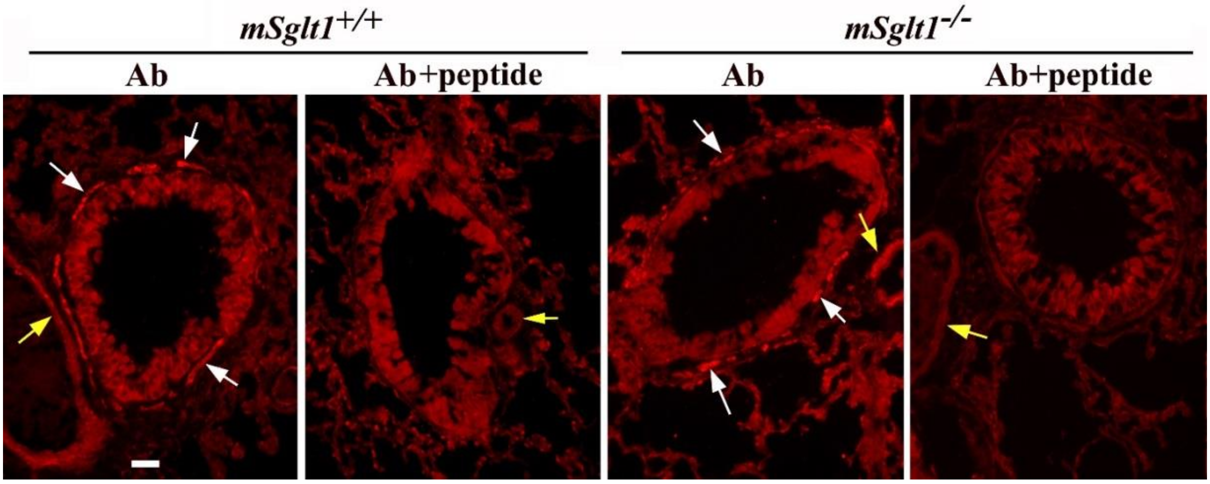

Fig. S3. Immunoreactivity of mSglt1-Ab in the lung of *mSglt1*$^{+/+}$ and *mSglt1*$^{-/-}$ mice without and with preabsorption of mSglt1-Ab with antigenic peptide. In *mSglt1*$^{+/+}$ mice, mSglt1-Ab stained the myoepithelial cells around bronchioli (white arrows) and with heterogeneous intensity the wall of arteries (yellow arrow). Both structures were similarly stained in *mSglt1*$^{+/+}$ and *mSglt1*$^{-/-}$ mice. Staining in all locations was abolished when mSglt1-Ab had been preabsorbed with the antigenic peptide (Ab+peptide). The methods are described in the main paper. Bar, 20 µm for all images. The data are representative for similar findings in 3 male and 3 female mice of each genotype.

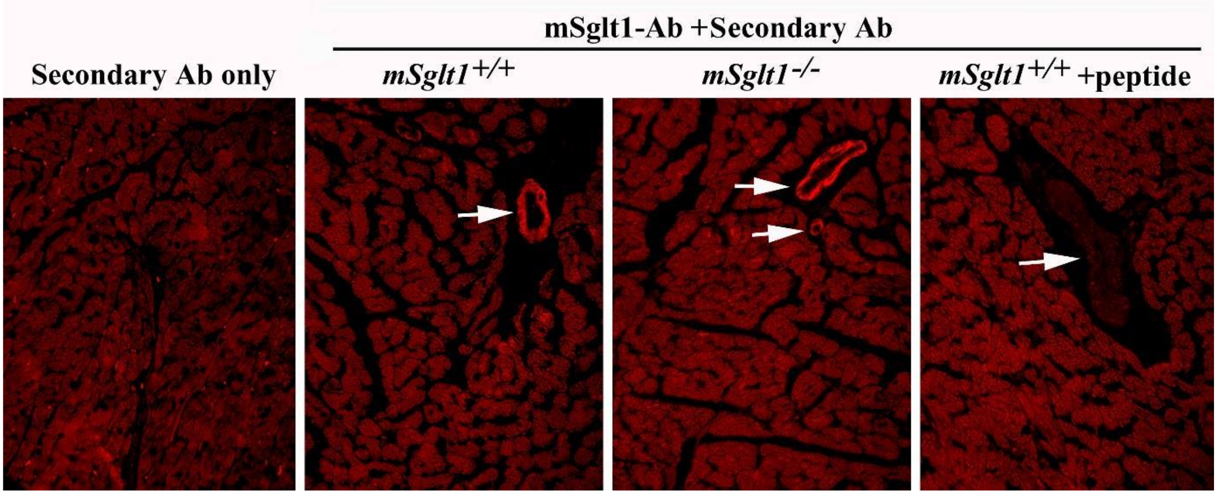

Fig. S4. Immunoreactivity of mSglt1-Ab in the heart (ventricle muscle) of *mSglt1*$^{+/+}$ and *mSglt1*$^{-/-}$ mice without and with preabsorption of mSglt1-Ab with antigenic peptide. In both *mSglt1*$^{+/+}$ and *mSglt1*$^{-/-}$ male and female mice, mSglt1-Ab stained with heterogeneous intensity the wall of some arteries (arrows). However, in the tissues from both genotypes many arteries remained unstained (not shown). When present, this staining was abolished when mSglt1-Ab had been preabsorbed with the antigenic peptide (Ab+peptide, arrow). The secondary antibody alone only nonselectiv background staining was observed which is probably due to autofluorescence. The methods are described in the main paper. Bar, 20 µm for all images. The data are representative for the findings in heart tissue from 3 male and 3 female mice of each genotype.

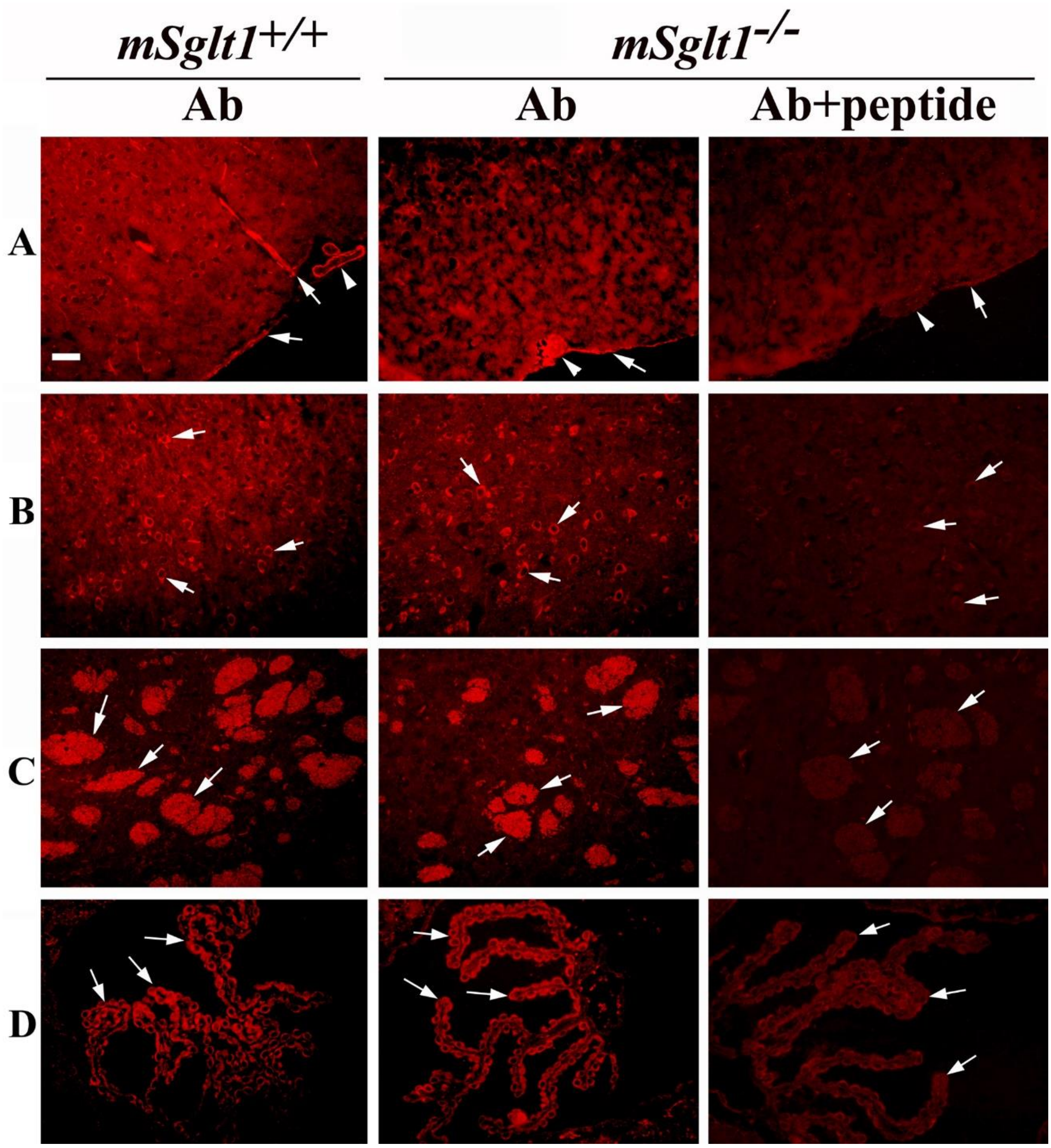

Fig. S5. Immunostaining of sections from cerebrum with mSglt1-Ab in *mSglt1*$^{+/+}$ and *mSglt1*$^{-/-}$ mice. In *mSglt1*$^{+/+}$ mice, immunoreactivity of mSglt1-Ab (*mSglt1*$^{+/+}$, Ab) was observed in pia mater (A, arrows), arterial walls (A, arrowhead), pyramid cells (B, arrows), bunches of nerves (C, arrows), and epithelial cells of choroid plexus (D, arrows). The same structures were also stained in *mSglt1*$^{-/-}$ mice. In both, *mSglt1*$^{+/+}$ (not shown) and *mSglt1*$^{-/-}$ mice, the staining of these structures was abolished with the antibody that had been preabsorbed with the immunizing peptide (Ab+peptide). Similar, peptide-blockable staining in *mSglt1*$^{+/+}$ and *mSglt1*$^{-/-}$ mice (pia mater, arterial walls, neuronal bunches, Purkinje cells) was also detected in cerebellum (data not shown). Bar, 20 μm for all images. The data are representative for the findings in brain tissue from 3 male and 3 female mice of each genotype.

Immunoreactivity of commercial antibodies in the kidney and small intestine of mSglt1$^{+/+}$ and mSglt1$^{-/-}$ mice (Figs. S6-S8)

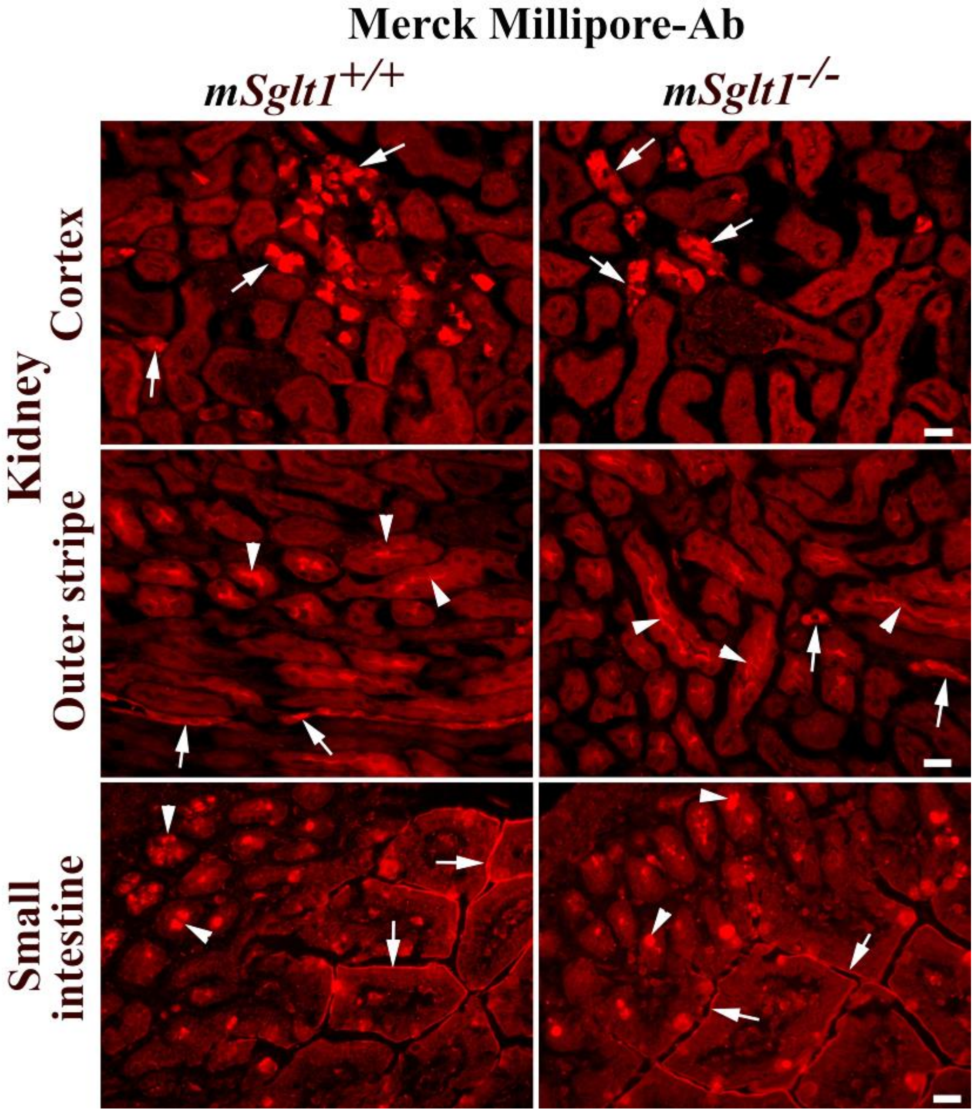

Fig. S6 Evaluation of the specificity of immunostaining with antibody #07-1417 from Merck Millipore (Merck Millipore-Ab) in kidney cortex and outer stripe, and in small intestine (duodenum) employing *mSglt1*$^{-/-}$ mice as specificity control. In both *mSglt1*$^{+/+}$ and *mSglt1*$^{-/-}$ mice similar staining was observed in each organ. The non-identified cells in collecting ducts were stained in renal cortex and outer stripe (arrows). In outer stripe, also the luminal domain of S3 segments was stained (arrowheads). In small intestine, the antibody stained the brush-border of enterocytes (arrows) and various non-identified cells in the stroma (arrowheads). Bars, 20 μm. The methods are described in the main paper. The data represent the findings in the organs from 3 male mice of each genotype. The data indicate that this antibody is not specific for mSglt1. Apparently, it reacts with a protein which has overlapping locations with mSglt1.

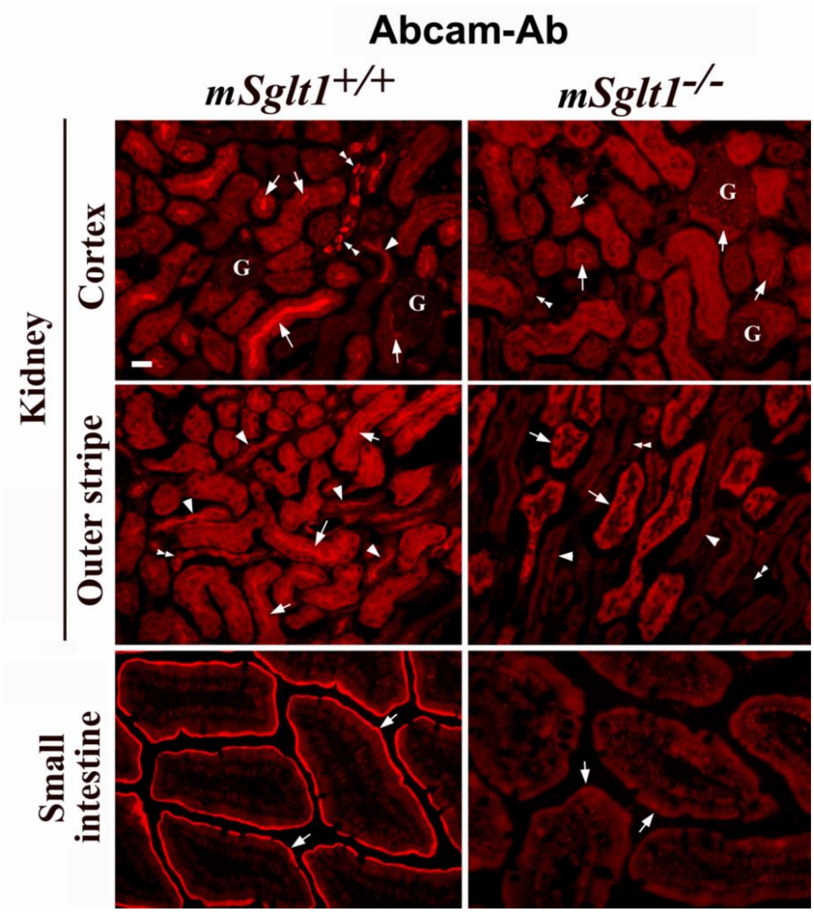

Fig. S7. Evaluation of the specificity of immunostaining with antibody #14686 from Abcam (Abcam-Ab) in kidney cortex and outer stripe, and in small intestine (duodenum) employing *mSglt1*$^{-/-}$ mice as control for specificity. In the kidney cortex of *mSglt1*$^{+/+}$ mice, luminal staining of proximal convoluted tubules (arrows) and thick ascending limb of Henle (TALH; arrowheads) was observed. In addition, non-identified cells in collecting ducts (double small arrowheads) were brightly stained. In the cortex of *mSglt1*$^{-/-}$ mice, the staining of proximal convoluted tubules was reduced but not abolished (arrows), whereas the staining in TALH (not shown) and collecting duct cells (double small arrowheads) was absent. In the outer stripe of *mSglt1*$^{+/+}$ mice, weak luminal staining of proximal tubule S3 segments (arrows) and TALH (arrowheads), and the staining of collecting duct cells (double small arrowheads) was also detected, whereas in *mSglt1*$^{-/-}$ mice, these structures were unstained. In small intestine (duodenum), the brush-border of enterocytes (arrows) was strongly stained in *mSglt1*$^{+/+}$ mice, whereas no staining was observed in *mSglt1*$^{-/-}$ mice. The methods are described in the main paper. G, glomeruli. Bar, 20 µm for all images. The data represent the findings in the organs from 3 male mice of each genotype. The data indicate that this antibody works nicely in small intestine, whereas in the mouse kidney, it reacts weakly with mSglt1 and shows nonspecific reactions with collecting ducts.

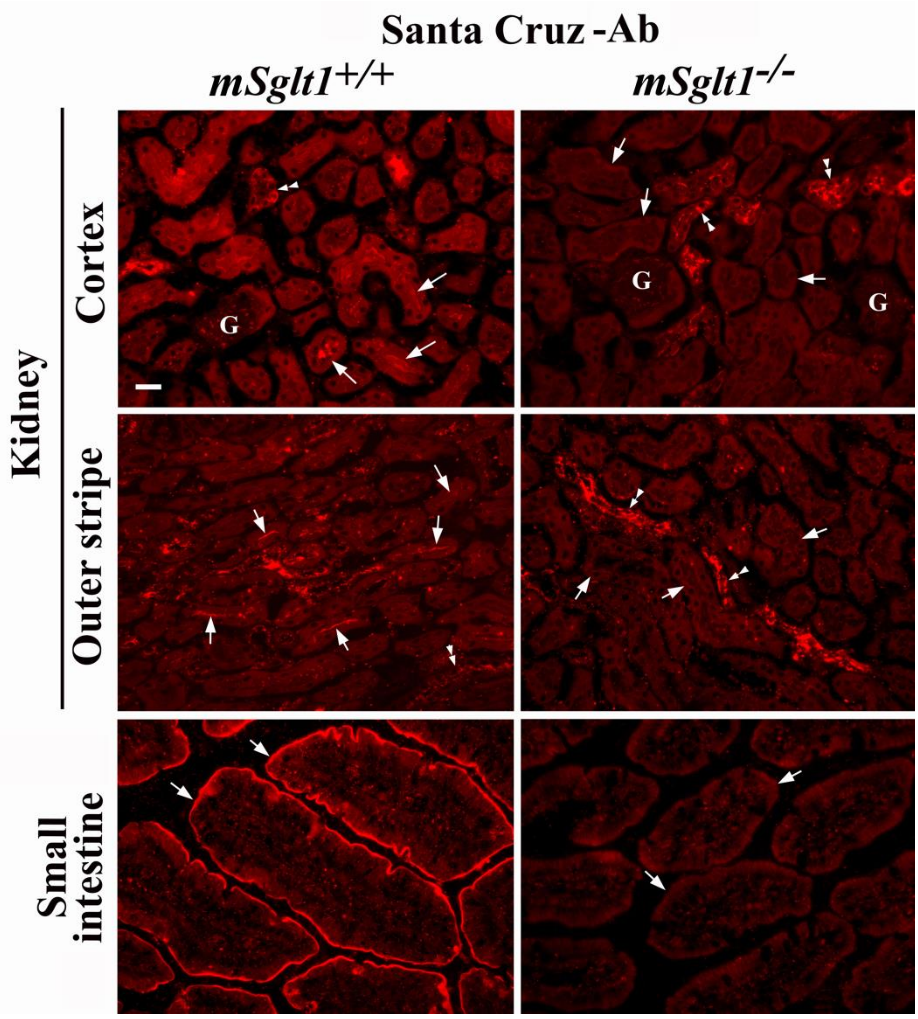

Fig. S8. Evaluation of the specificity of immunostaining with antibody sc-20582 from Santa Cruz (Santa Cruz-Ab) in kidney cortex, outer stripe, and small intestine (duodenum) employing *mSglt1*$^{-/-}$ mice as control for antibody specificity. In the kidney cortex of *mSglt1*$^{+/+}$ mice, the antibody weakly stained the luminal domain of proximal tubules (arrows) and some collecting duct cells (double small arrowheads). In *mSglt1*$^{-/-}$ mice, proximal tubules were unstained (arrows), while the collecting duct cells remained stained (double small arrowheads). In the outer stripe of *mSglt1*$^{+/+}$ mice, the antibody weakly stained the luminal membrane of some, but not all S3 segments (arrows), and the collecting duct cells (double small arrowhead). In *mSglt1*$^{-/-}$ mice, S3 segments in the outer stripe were unstained, whereas the collecting duct cells remained stained. In the small intestine of *mSglt1*$^{+/+}$ mice, the brush-border of enterocytes was strongly stained (arrows), and this staining was absent in *mSglt1*$^{-/-}$ mice. The methods are described in the main paper. G glomeruli. Bar, 20 µm (for all images). The data are representative for the findings in the organs from 3 male mice of each genotype. The data indicate that this antibody is specific for small intestine, but not good for studies in mouse kidney.

Immunoreactivity of commercial anti-SGLT1 antibodies in the lung (Fig. S9), heart (Fig. S10) and brain (Fig. S11) of mSglt1$^{+/+}$ and mSglt1$^{-/-}$ mice

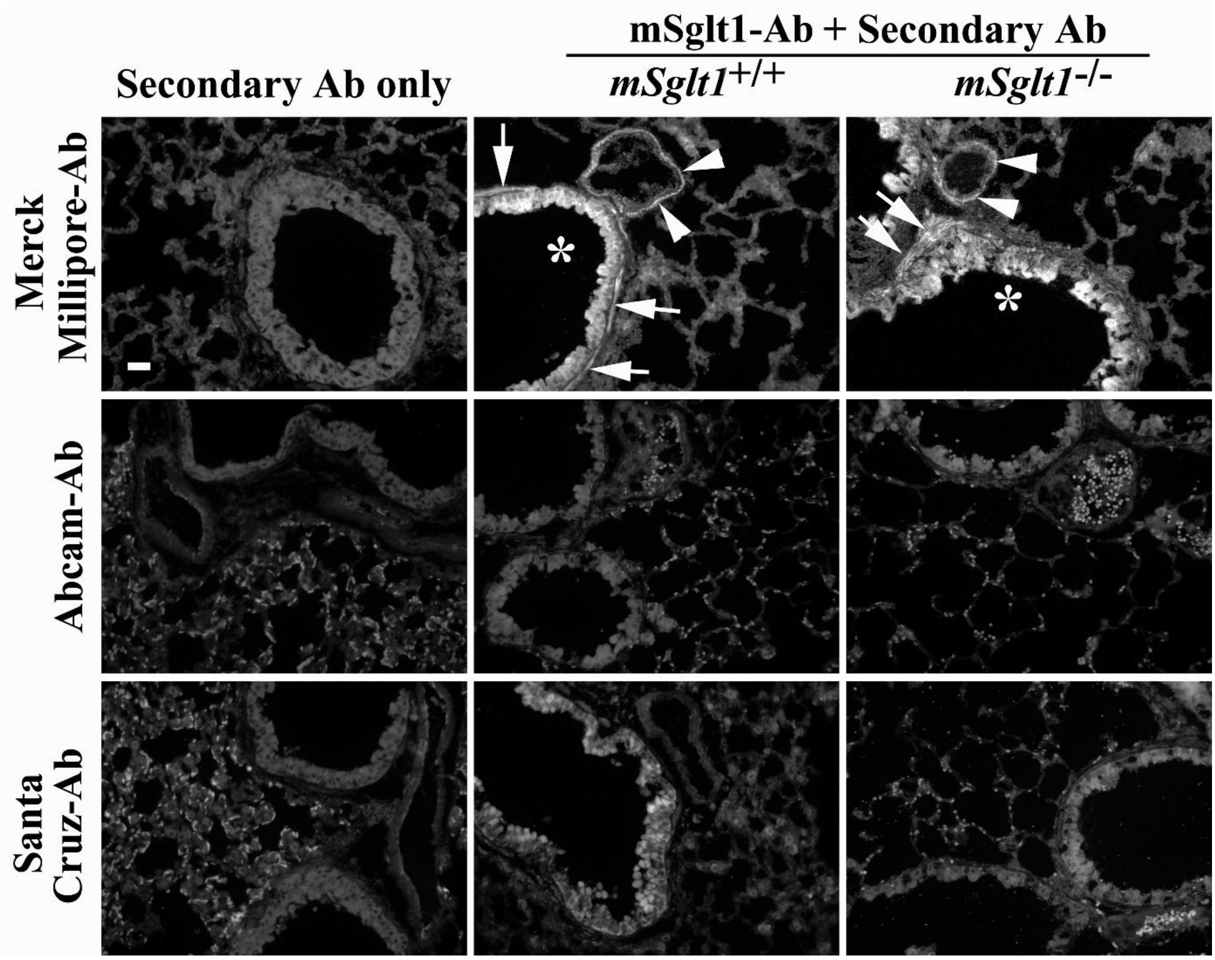

Fig. S9 Immunoreactivity of the commercial anti-SGLT1 antibodies in lung of *mSglt1*$^{+/+}$ and *mSglt1*$^{-/-}$ mice. With secondary antibody alone some nonselective background staining was observed which is probably due to autofluorescence. With Merck Millipore-Ab similar staining of bronchial epithelial cells (asterisk), peribronchial myoepithelial cells (arrows), and the wall of arteries (arrowheads) was observed in both *mSglt1*$^{+/+}$ and *mSglt1*$^{-/-}$ mice. Apparently this staining is not related to mSglt1. With Abcam-Ab and Santa Cruz-Ab, only a background staining was observed. Red-stained images were converted to black and white using the Photoshop program in order to improve contrast. The methods are described in the main paper. Bar, 20 µm for all images. The data are representative for the findings in the lung tissue from 3 male and 3 female mice of each genotype.

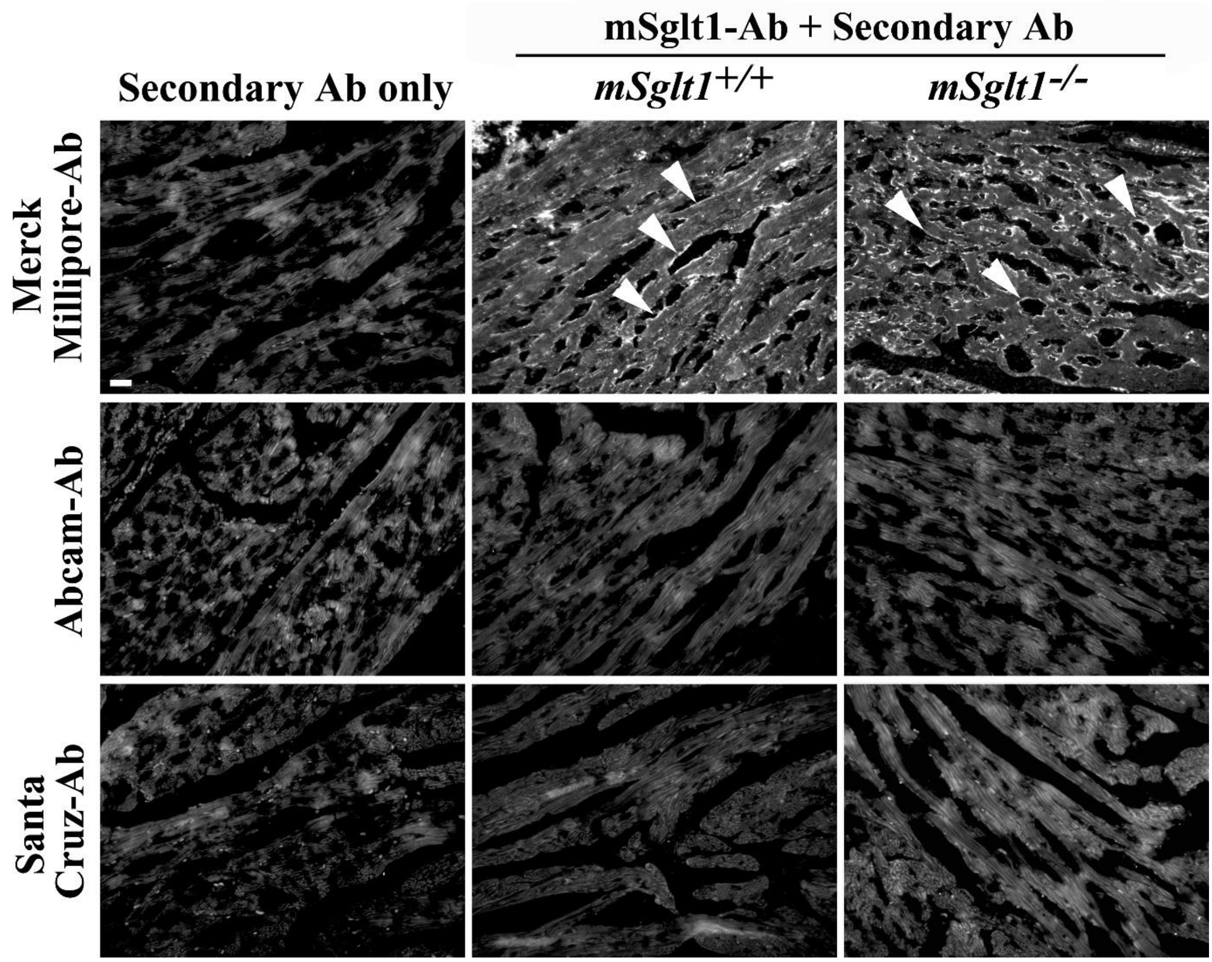

Fig. S10. Immunoreactivity with commercial anti-SGLT1 antibodies in the heart ventricle of *mSglt1*$^{+/+}$ and *mSglt1*$^{-/-}$ mice. With secondary antibodies only nonselective background staining was observed. Merck Millipore-Ab strongly stained the surface of cardiocytes in *mSglt1*$^{+/+}$ mice, mimicking the staining of sarcolema (arrowheads). However, identical staining was observed in the heart of *mSglt1*$^{-/-}$ mice, indicating nonspecificity. Abcam-Ab and Santa Cruz-Ab exhibited no immunoreactivity in the heart of both mouse genotypes. Red-stained images were converted to black and white as in Fig. S9. The employed methods are described in the main paper. Bar, 20 µm for all images. The data are representative for the findings in the heart tissue from 3 male and 3 female mice of each genotype.

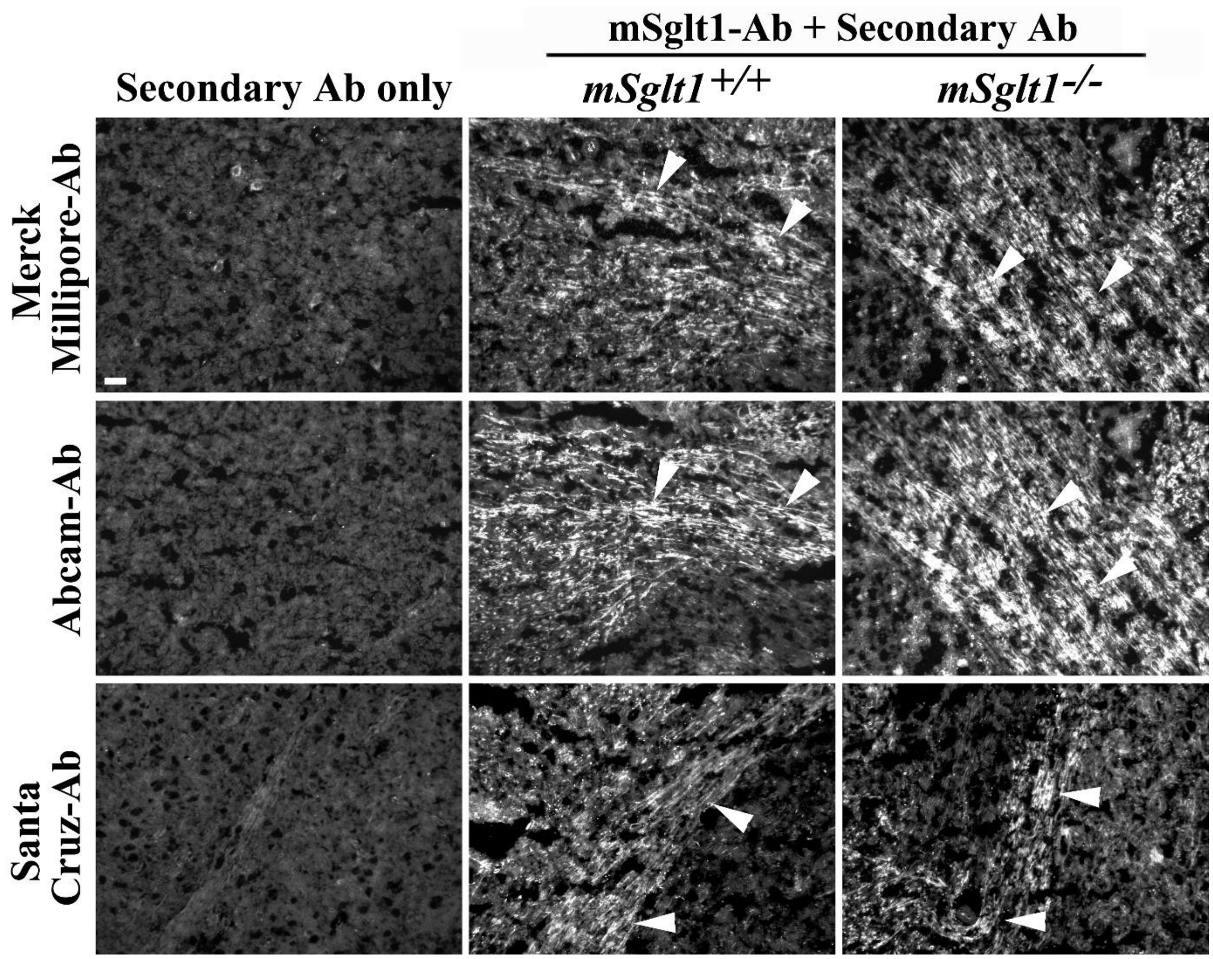

Fig. S11. Immunostaining of cryosections from cerebrum with commercial anti-SGLT1 antibodies in *mSglt1*$^{+/+}$ and *mSglt1*$^{-/-}$ mice. With secondary antibodies alone only weak nonselective background staining was observed. In both *mSglt1*$^{+/+}$ and *mSglt1*$^{-/-}$ mice bunches of nerves were strongly stained with all three antibodies (arrowheads) indicating nonspecific immunoreactivity. Red-stained images were converted to black and white as in Fig. S9. Bar, 20 μm for all images. The data are representative for the findings in the brain tissue from 3 male and 3 female mice of each genotype.
